# Human antibody targeting of coronavirus spike S2 subunit is associated with protection mediated by Fc effector functions

**DOI:** 10.1128/jvi.01523-25

**Published:** 2025-11-12

**Authors:** Krithika Muthuraman, Matthew Jackman, Yu Liang, Meghan E. Garrett, Hong Cui, Loan Vu Hong Nguyen, Danton Ivanochko, Chengjin Ye, Paula A. Pino, Amberlee Hicks, Billie Maingot, Erik Yusko, Sharon Benzeno, Luis Martínez-Sobrido, Jordi B. Torrelles, Amy E. Gilbert, Benjamin Evan Russell Rubin, Gladys Keitany, Arif Jetha, Jean-Philippe Julien

**Affiliations:** 1 Program in Molecular Medicine, The Hospital for Sick Children Research Institute 7979, Toronto, Ontario, Canada; 2 Department of Biochemistry, University of Toronto 7938, Toronto, Ontario, Canada; 3 Adaptive Biotechnologies 478252 , Seattle, Washington, USA; 4 Population Health Program, Texas Biomedical Research Institute 7075, San Antonio, Texas, USA; 5 International Center for Advancement of Research & Education (I· CARE), San Antonio, Texas, USA; 6 Department of Immunology, University of Toronto 7938, Toronto, Ontario, Canada; St Jude Children's Research Hospital, Memphis, Tennessee, USA

**Keywords:** cryo-EM structure, *in vivo* protection, S2 subunit, coronavirus spike, antibody

## Abstract

Over the past two decades, betacoronaviruses (β-CoVs) have caused two epidemics and a pandemic and remain a high risk for future outbreaks through zoonotic transmissions, highlighting the need for broad biomedical countermeasures. Here, we describe a convalescent human monoclonal antibody (mAb 1871) that targets the S2 subunit of the coronavirus spike protein, with broad β-CoVs binding and sarbecovirus neutralization. Cryo-electron microscopy analysis revealed that mAb 1871 binds the upstream helix of the S2 subunit, interacting with partially conserved residues, providing a molecular basis for its cross-reactivity. Though less potent than receptor-binding domain-directed antibodies—approximately 500-fold lower neutralization potency than the emergency use authorized receptor-binding domain (RBD)-directed Pemgarda mAb against wild-type SARS-CoV-2—mAb 1871 provides protective efficacy in a mouse model. Notably, Fc effector functions are critical for its *in vivo* protection. This study further highlights the Fc dependence of S2-directed antibodies for *in vivo* protection and identifies a conserved epitope in the S2 subunit as a potential target of broad-β-CoVs countermeasures.

**IMPORTANCE:**

Bats and pangolins are natural reservoirs of betacoronaviruses (β-CoVs) and continue to pose a significant risk for future outbreaks through zoonotic transmissions. This highlights the need for effective countermeasures to prevent future pandemics. While neutralizing antibodies targeting the receptor-binding domain of severe acute respiratory syndrome coronavirus 2 (SARS-CoV-2) received emergency use authorization, many have lost efficacy as the virus evolved, and authorizations have been revoked. In contrast to the S1 subunit, the spike protein S2 subunit is more conserved across β-CoVs, making it an attractive target for the development of broadly neutralizing antibodies. Here, we describe a human mAb that targets a conserved epitope in the S2 subunit, demonstrating broad β-CoV binding, sarbecovirus neutralization, and *in vivo* protection mediated by Fc effector functions in a mouse model. These findings have important implications for pan-β-CoVs therapeutics and vaccine development.

## INTRODUCTION

Severe acute respiratory syndrome coronavirus 2 (SARS-CoV-2) caused the COVID-19 pandemic, which presented an enormous economic and public health challenge. SARS-CoV-2 belongs to the family of *Coronaviridae* and the genus Betacoronavirus. Betacoronaviruses (β-CoVs) are one of the four genera of coronavirus (alpha, beta, gamma, and delta), with a large 30 kbp single-stranded (ss)RNA, that usually cause mild to moderate upper-respiratory tract infections. Previous coronavirus zoonotic transmissions have caused widespread infections and led to the circulation of four endemic human coronaviruses e.g., HCoV-229E, HCoV-NL63 (belonging to alphacoronavirus genus), HCoV-OC43, and HCoV-HKU1 (belonging to β-CoVs genus) that cause non-severe seasonal respiratory infections (1). However, in the last two decades, β-CoVs have caused two epidemics and a pandemic, namely SARS (2002), Middle Eastern respiratory syndrome (MERS) (2012), and COVID-19 (2019), with high lethality. Bats and rodents are natural reservoirs of β-CoVs, posing a continued high risk for future outbreaks through zoonotic transmissions (2, 3). Therefore, to ensure pandemic preparedness, the swift development of broad biomedical countermeasures against emerging β-CoVs is paramount.

Pathogenesis of β-CoVs begins with receptor binding via the trimeric spike surface glycoprotein, followed by enzymatic cleavage and a conformational change in the spike, leading to fusion of viral and cell membranes. Spike protein (SP) consists of two subunits, namely, the S1-subunit consisting of the receptor-binding domain (RBD) and the S2-subunit containing the fusion machinery. SP is an immunodominant target for antibodies (Abs). RBD-directed Abs can block receptor binding and are the most potent; hence, these have been the major focus of therapeutic Abs and vaccine discovery efforts. However, the emergence of SARS-CoV-2 variants of concern (VOCs) (4) quickly rendered SARS-CoV-2 RBD-directed Ab interventions, such as the Bamlanivimab and Etesevimab cocktail (5), or the REGN-COV cocktail (6), ineffective to treat COVID-19, consequently losing their emergency use authorization. The loss in potency is due to a constellation of mutations within the SARS-CoV-2 SP, especially within the RBD (7). The S1 subunit exhibits less conservation among β-CoVs due to different use of entry receptors and because it is subject to significant immune pressure, which leads to increased mutations aimed at evading immune responses (8). In contrast, the S2 subunit shows a relatively higher conservation of 63%–98% (9) across different β-CoVs, which makes it an attractive target for the development of broad β-CoV-neutralizing Abs.

Several Abs targeting two conserved sites in the SARS-CoV-2 SP S2 subunit—the stem helix (10–14) and fusion peptide (15, 16)—isolated from humans and vaccinated animals display broad β-CoV neutralization profiles, although at lower potencies than RBD-directed Abs. Furthermore, these Abs are shown to provide *in vivo* protection despite relatively low *in vitro* neutralization potency, with this effect attributed to their dependence on Fc effector functions (10, 11). Identification of pan-β-CoVs Abs could aid in pandemic preparedness and rapid deployment in the event of an outbreak. Furthermore, information from these Abs that target novel conserved epitopes could be used for structure-based design of pan-β-CoV vaccines (17–19).

ADPT01871 (herein referred to as mAb 1871), a S2 subunit-directed Ab isolated from a COVID-19 convalescent donor, displayed broad β-CoV binding, SARS-CoV and SARS-CoV-2 neutralization, and blocked *in vitro* cell membrane fusion (20). Despite relatively low *in vitro* potency, mAb 1871 provided protection against SARS-CoV-2 in a mouse challenge study (20). Here, we sought to expand our understanding of the molecular basis of binding for mAb 1871. Indeed, previous efforts in epitope identification had proved challenging due to differences in prefusion and postfusion conformations, flexibility of target regions, and low affinity of this mAb. Here, we used cryo-electron microscopy (cryo-EM) for the full molecular characterization of mAb 1871 and delineated its epitope on the S2 subunit. We also further describe the importance of effector function for this SP S2-subunit directed mAb in providing *in vivo* protection.

## RESULTS

### mAb 1871 possesses broad betacoronavirus binding

**Fig 1:**
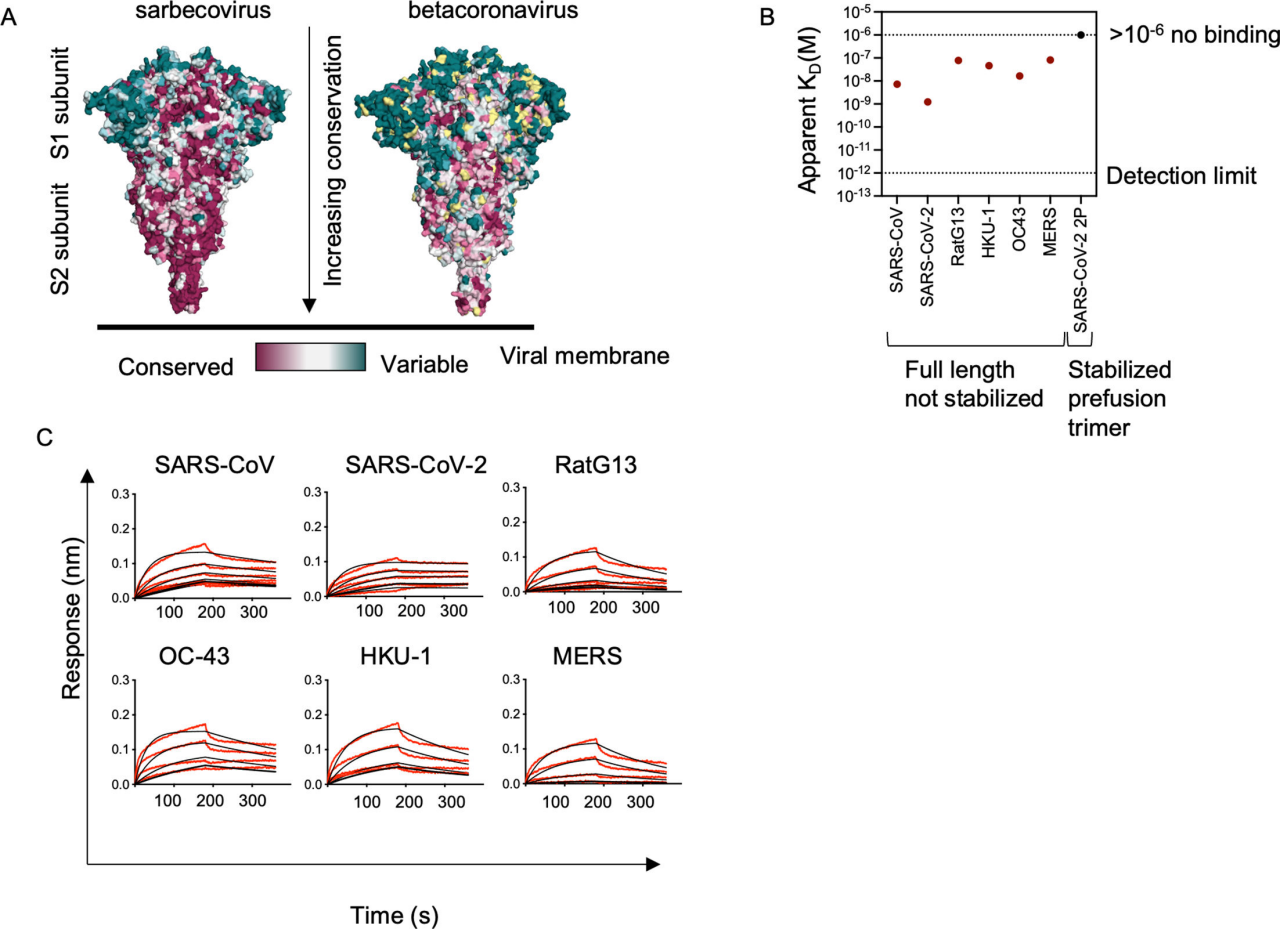
Broad β-CoV binding of mAb 1871. (**A**) Sequence conservation across the panel of β-CoV SP listed in Fig. 4B shown in a colored representation on SARS-CoV-2 SP PDB ID: 6XR8 (22). The left panel shows conservation across sarbecovirus sequences, and the right panel shows conservation across β-CoV SP. Pink indicates conserved residues and teal indicates variable residues. Amino acids with insufficient data are shown in yellow. Coloring generated using the Consurf server (23–28). Consurf mapping was generated based on sequence homology of the supplied model of SARS-CoV-2 spike, PDB ID: 6XR8 (22). (**B**) Comparison of apparent binding affinity (K_D_) of mAb 1871 to full-length SP (SARS-CoV, SARS-CoV-2, HKU-1, OC43, RatG13, and MERS) shown as red circles and SARS-CoV-2 prefusion stabilized trimer shown as a black circle. K_D_ values generated by global fitting from six different mAb concentrations from a single representative experimental data are depicted. (**C**) Sensograms showing binding kinetics of mAb 1871 to full-length SARS-CoV, SARS-CoV-2, HKU-1, OC43, RatG13, and MERS SP. Red lines represent raw data, and black lines represent global fit.

mAb 1871 was isolated from Ab-secreting cells of a 30-year-old male infected with SARS-CoV-2 in the acute phase of illness, 9 days post-symptom onset, using pairSEQ technology (20). mAb 1871 can bind a broad panel of β-CoV full-length SP (SARS-CoV, SARS-CoV-2, MERS-CoV, HCoV-HKU1, and HCoV-OC43) and SP S2 subunits, and can neutralize SARS-CoV, SARS-CoV-2 wild-type USA/WA1/2020, alpha and beta VOC pseudoviruses with IC_50_s of 30, 0.44, 0.11, and 0.21 nM, respectively (20). This broad binding and neutralization profile suggests that mAb 1871 may be directed against one of the conserved epitopes in the S2 subunit domain, where a high sequence conservation is observed among β-CoVs (Fig. 1A) (9). To investigate whether mAb 1871 binding is dependent on SP conformation, we interrogated binding to full-length SP (SARS-CoV, SARS-CoV-2, RatG13, MERS, HKU1, OC43), full-length SP trimer stabilized in the prefusion conformation (SARS-CoV-2 SP full length with a polybasic cleavage site deletion and stabilizing mutations K986P and V987P, wild-type numbering [21]), and S2 subunit-only (SARS-CoV, SARS-CoV-2, MERS, OC43) proteins by biolayer interferometry. mAb 1871 bound to the full-length SP β-CoVs panel with an apparent K_D_ in the range of 0.1 to 7 nM (Fig. 1B and C). mAb 1871 also bound to S2 subunit domains (Fig. S1). However, binding was not observed to the prefusion stabilized full-length SARS-CoV-2 SP trimer (Fig. 1B; Fig. S1), thereby suggesting that the recognized epitope may not be available in the prefusion state and might only become exposed after a conformational change occurs.

**Fig 2:**
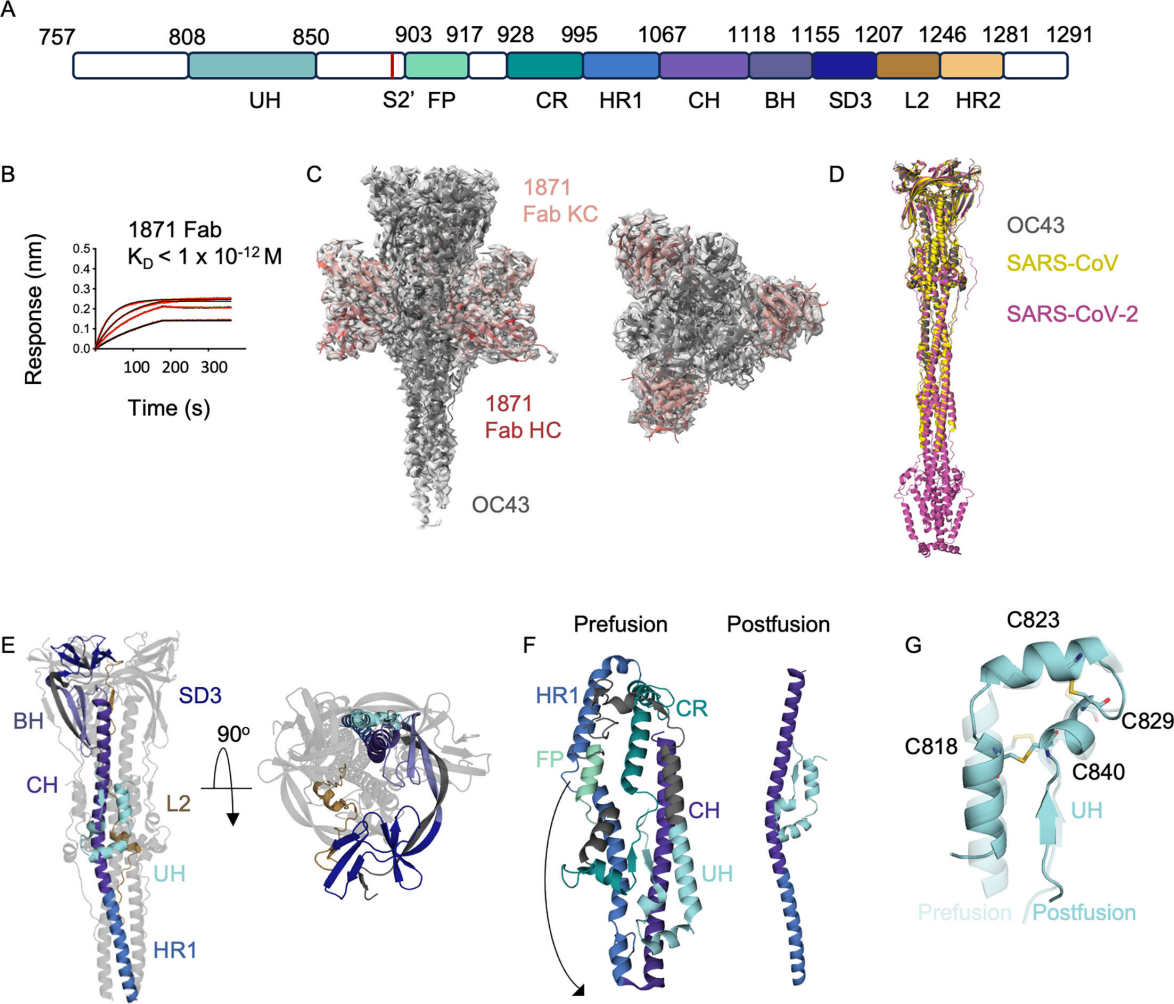
Structural disposition of OC43 S2 subunit. (**A**) Schematic diagram of OC43 SP S2 subunit ectodomain—Upstream helix (UH), fusion peptide (FP), Connecting region (CR), heptad repeat 1 (HR1), central helix (CH), beta hairpin (BH), subdomain 3 (SD3), linker 2 (L2), and heptad repeat 2 (HR2) depicted in different colors. (**B**) Sensogram of 1871 Fab binding to OC43 S2 subunit. K_D_ values generated by global fitting from four different Fab concentrations from a single representative experimental data are depicted. (**C**) Cryo-EM 3D reconstruction of 1871Fab-OC43 S2 complex at 2.6 Å resolution. Side and top view. (**D**) Comparison of postfusion structures of OC43 colored in gray, along with SARS-CoV (29) (PDB ID: 6M3W) colored in yellow and SARS-CoV-2 (30) (PDB ID: 8FDW) colored in pink. (**E**) OC43 S2 subunit domains in postfusion conformation. Side and top view. (**F**) Comparison of prefusion (PDB ID: 7PNM) (31) OC43 S2 domain showing conformational changes leading to movement of heptad repeat to form a long alpha helical structure in the postfusion conformation. (**G**) Comparison of the upstream helix in prefusion conformation (PDB ID: 7PNM) (31) shown in transparent cyan and post-fusion conformation shown in cyan. Disulfide bonds are depicted in yellow.

Although mAb 1871 was isolated only 9 days post-symptom onset, a high occurrence of somatic hypermutations (SHM) was observed (20). Interestingly, the fragment antigen-binding (Fab) 1871 displayed high binding affinity to OC43 S2 subunit (Fig. 2A and B) compared to MERS, SARS-CoV, and SARS-CoV-2 (Fig. S2), suggesting that this mAb might have initially developed during an infection by this commonly encountered β-CoV.

### OC43 S2 subunit adopts an elongated postfusion conformation

We used cryo-EM to understand the molecular basis of S2 subunit recognition by Fab 1871. For this purpose, we focused on the high-affinity OC43 S2 subunit construct comprising residues 757–1,291 along with a *C*-terminal T4 foldon trimerization domain (Fig. 2A). Analysis of cryo-EM micrographs and 2D classes revealed three Fabs bound to an elongated structure reminiscent of the post-fusion SP of SARS-CoV-2(22), SARS-CoV (29), and MHV(32) (Fig. S3). Since most particles observed tended to have an orientation bias (Fig. S3J), data were collected at a 40° tilt to improve the distribution of orientations (Fig. S4). When C3 symmetry was applied, the single particle reconstructions of the Fab-S2 subunit complex generated a map at a resolution of 2.6 Å (Fig. 2C; Fig. S3 and S4 Table S1).

The cryo-EM reconstruction revealed that the OC43 S2 subunit adopts a similar structure to the postfusion conformation of both SARS-CoV-2(30) (PDB ID: 8FDW, rmsd = 1.1 Å) and SARS-CoV (29) (PDB ID: 6M3W, rmsd = 1.2 Å) (Fig. 2D), corroborating high sequence similarity among these β-CoV strains (Fig. S5). The regions resolved in the OC43 structure include almost all domains; linker-1 (L1; 783–808), upstream helix (UH; 809–850), heptad repeat 1 (HR1; 995–1,067), central helix (CH; 1,068–1,118), beta hairpin (BH; 1,119–1,155), sub-domain 3 (SD3; 11,56–1,207), linker-2 (L2; 1,208–1,246), but not the fusion peptide (FP; 903–917), connecting region (CR; 928–995), and heptad repeat 2 (HR2; 1,247–1,281) (Fig. 2E).

As previously reported for other postfusion SP structures (29, 30), the core of OC43 S2 subunit forms a conical shape that is approximately 135 Å long and 50 Å wide, having a long three-helix bundle formed by CH and HR1, with a three-stranded β-sheet wrapping around the C-terminal end of the CH bundle (Fig. 2E). In the prefusion conformation, the S2 subunit is short and then undergoes a large conformational change during which the HR1 and connecting loops re-orient with the central helix to form a single long continuous alpha-helix (Fig. 2F, Fig. S6A). This forms the stable triple helix bundle of the post-fusion S2 subunit core. Four disulfide bonds in the structure, two within the UH (C816–C838, C821–C827), one in BH (C1116–C1126), and one between the SD3 and L2 (C1166–C1211) are conserved both in the pre-fusion and post-fusion conformations (Fig. 2F and G; Fig. S6B). These disulfide bonds help the UH, BH, and SD3 domains retain their tertiary structure between the two conformational states.

### mAb 1871 binds to the upstream helix in the S2 subunit

**Fig 3:**
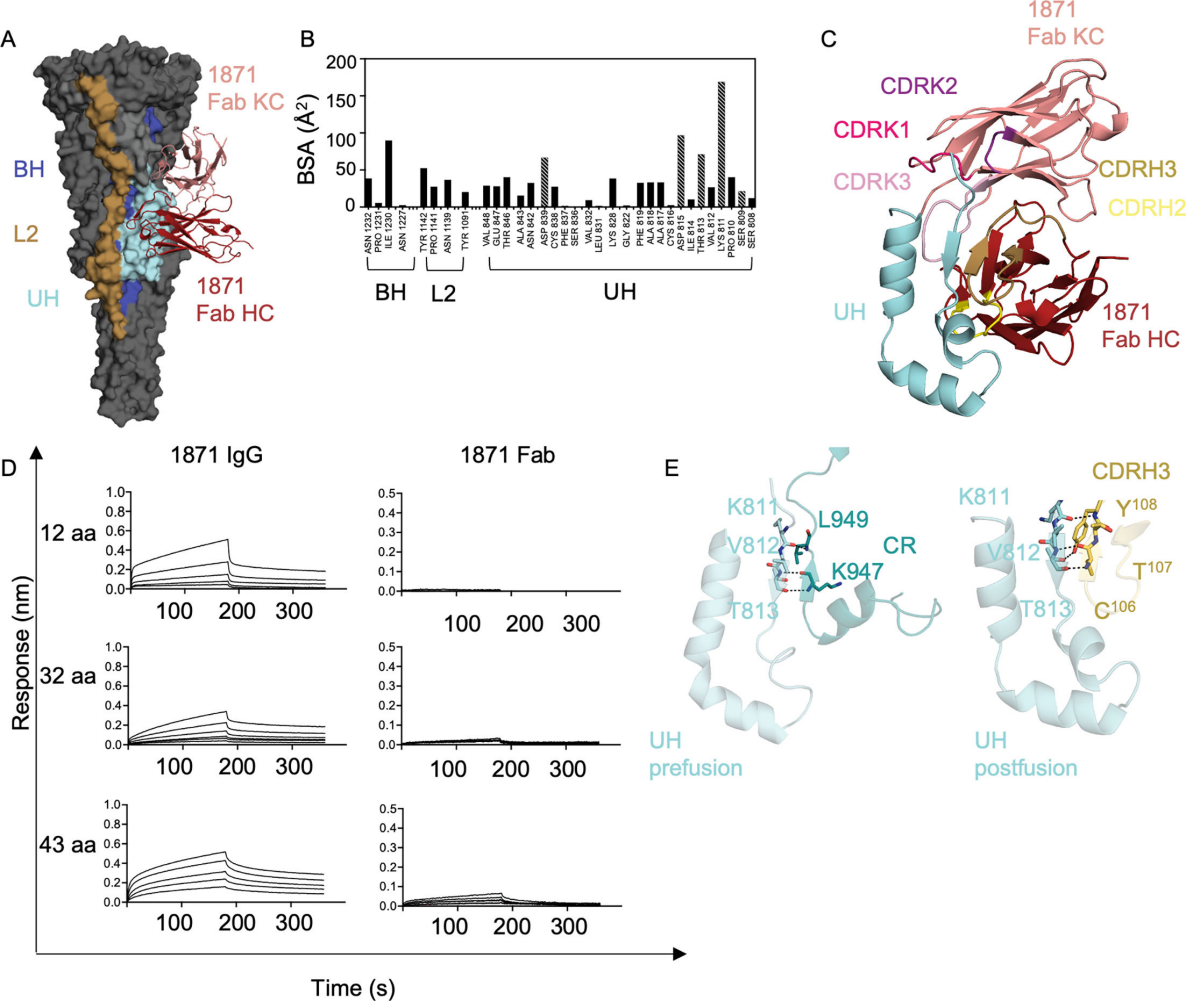
Structural details of mAb 1871 binding. (**A**) 1871 epitope. OC43 S2 subunit colored in gray. 1871 epitope—UH shown in cyan, BH shown in purple, and L2 shown in brown. 1871 heavy chain shown in red and light chain shown in salmon. (**B**) BSA of interface residues in OC43 S2 subunit. Shaded bars indicate residues involved in hydrogen bonding, and solid bars indicate residues that are not involved in hydrogen bonding. (**C**) UH shown in cyan, 1871 heavy chain in dark red, and light chain in salmon. CDRs involved in the interaction are represented in dark yellow (CDRH3), light yellow (CDRH2), magenta (CDRK1), and light pink (CDRK3). (**D**) Sensograms depicting binding profiles of 1871 IgG and Fab to 12 aa, 32 aa, and 43 aa upstream helix peptides. (**E**) Anti-parallel beta strand interaction and hydrogen bonds involved between UH and CR in pre-fusion OC43 (PDB ID: 7PNM) (31) and post-fusion UH and CDRH3 of 1871. Residues involved in hydrogen bonds are depicted as sticks, and hydrogen bonds are depicted as dashed black lines.

To gain further confidence in the mAb 1871 structure, the unliganded 1871 Fab was solved by X-ray crystallography to 2.5 Å resolution (Table S2) and used as a starting model to build in the cryo-EM map. The low rsmd value of unliganded and liganded Fab structures for the variable region heavy (0.3 Å) and light chain (0.3 Å) suggests that the antigen and Ab largely interact via a lock and key mechanism. Each 1871 Fab interacts with two OC43 S2 subunit protomers via a conformational epitope consisting of UH, BH, and L2 (Fig. 3A; Fig. S6D), with UH contributing the majority of the interface with 25 interacting residues (Fig. 3B). The buried surface area (BSA) of the S2 subunit in the interface is 1,024 Å^2^, with 541 Å^2^ conferred by the Ab heavy chain and 483 Å^2^ conferred by the light chain. Residues from heavy chain complementarity-determining regions (CDRs) H2 and H3, light chain CDRs K1, K2, and K3, along with four residues in the kappa chain framework, form part of the interface (Fig. 3C). The four framework residues of kappa chain—Y49, I52, G64, and S65—interact with BH. Of these, residue I52 is mutated from the germline IGKV3-15*01 residue (T52I). Eight residues in the UH interact with seven residues in the heavy chain CDRs H2 and H3, and five residues in the light chain CDRs K1 and K3 via an extensive network of 18 hydrogen bonds (Fig. 3C; Table S3). Interfacing residues in L2 and BH do not contribute any hydrogen bond or salt bridge interactions. To further validate the structure interface, we performed binding to UH peptides of varying lengths. IgG bound to all three peptides of 12, 32, and 43 amino acids, whereas the Fab showed very weak binding only to the 43 amino acid peptide at the highest Fab concentration (Fig. 3D). Notably, the peptides contain different interface residues involved in the interaction, with the 12-aa peptide containing 5 hydrogen bonding residues, the 32-aa peptide containing 6 hydrogen bonding residues, and the 43-aa peptide containing all relevant residues. This suggests that, due to avidity, the IgG can bind to an incomplete epitope (shorter peptides), whereas the Fab, lacking avidity, displays weak binding only to a near-complete epitope (42-aa peptide). However, when the full epitope is presented, in the context of the S2 subunit, as observed in the structure, the Fab binds with high affinity.

Furthermore, the CDRH3 of 1871 Fab forms an anti-parallel β-sheet with the UH residues 811–815 via interaction through three hydrogen bonds (Fig. 3E). In the prefusion conformation (PDB ID: 7PNM) (31), hydrogen bond interactions between antiparallel β-strands in the CR and UH stabilize the region. Upon conformational change, the CR, along with the fusion peptide, moves toward the *C*-terminus of the postfusion structure. During this conformational change, the epitope on UH becomes available for 1871 binding, in a way that stabilizes the UH region in a similar way to the CR interaction (Fig. 3E). These structural insights help to better understand why 1871 does not bind to the prefusion stabilized SARS-CoV-2 trimer but binds to the S2 subunit (Fig. S1), as the stabilizing mutations lock the SP in its prefusion conformation, preventing movement in the CR. Furthermore, the UH region in the prefusion and postfusion conformations of OC43 has an rmsd value greater than 1.5 Å, indicating that structural differences may inhibit binding (Fig. S6C). S2-directed antibodies like S2P6 have been shown to bind both prefusion and postfusion S conformations, and their likely mechanism of action involves impeding fusogenic rearrangements (10). On the other hand, 1871 does not bind prefusion stabilized S (Fig. 1B; Fig. S1), and the resolved structure shows 1871 bound to a postfusion conformation (Fig. 2C). Together with functional assays demonstrating the ability of 1871 to block cell fusion (20), we propose the possibility that binding by 1871 to its UH epitope may also impede fusogenic rearrangements.

As structural analysis of SARS-CoV-2 spike in complex with 1871 Fab would provide the most relevant information pertaining to functional data, we performed Alphafold 3 (AF3) modeling (33). Modeling of the SARS-CoV-2 spike UH region with 1871 Fab revealed a binding profile similar to OC43 UH with 1871 Fab (Fig. S7A). Alignment of a single protomer from the AF3 SARS-CoV-2 model with the OC-43 upstream helix region provided an rmsd of 0.76 Å (Fig. S7B). Interface analysis using PDB PISA showed that the predicted interfacing residues between CoV-2 and 1871 were largely consistent with those seen in the OC43 complex, including several key hydrogen bonds (Fig. S7C).

### Upstream helix epitope is partially conserved across β-CoVs

**Fig 4:**
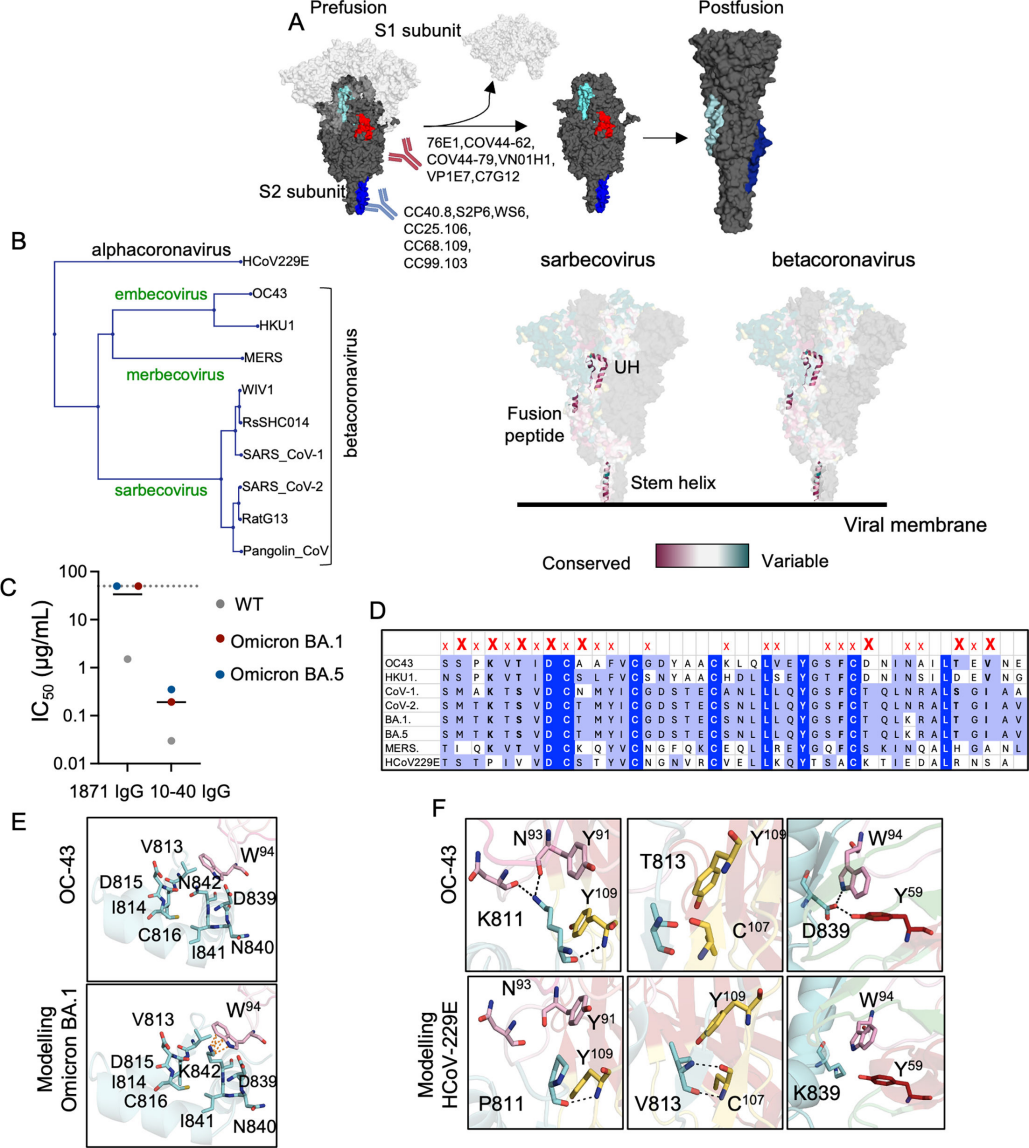
Structural analysis of sequence conservation in UH epitope. (**A**) Representation of conformational changes in the spike after receptor binding. Representation of SARS-CoV-2 SP with S1 subunit shown in light gray and S2 subunit shown in dark gray. Stem helix epitope colored in blue, fusion peptide in red, and upstream helix in cyan. Abs directed against the stem helix and fusion peptide are indicated. (**B**) Conservation analysis using consurf (23–28) for indicated spike sequences in the left panel for the three S2 subunit epitopes: stem helix, fusion peptide, and upstream helix. Pink indicates conserved residues and teal indicates variable residues. (**C**) Neutralization potency of mAbs 1871 and 10–40 (34, 35) RBD-directed IgG control against SARS-CoV-2 US/WA-1/2020 shown in gray, Omicron BA.1 shown in red, and Omicron BA.5 shown in blue. Mean IC_50_ from two biological replicates, each consisting of two technical replicates, is shown for each. (**D**) Sequence conservation in UH epitope across selected betacoronaviruses, including Omicron BA.1 and BA.5 VOC and an alphacoronavirus HCoV-229E. Sequences colored based on the BLOSUM62 score of percentage identity. X’s indicate interface residues, and bold X’s indicate residues involved in hydrogen bonding. (**E**) Molecular modeling indicates the possible conformation adopted by side chains of mutated residue in Omicron BA.1 and clashes are indicated by orange dashed lines. (**F**) Molecular modeling indicates possible conformations adopted by side chains of mutated residues in HCoV-229E, and hydrogen bonds are indicated as black dashed lines.

Known β-CoV-neutralizing Abs directed against the SARS-CoV-2 SP S2 subunit target two major epitope bins: the stem helix (1,140–1,160) and the fusion peptide (840–852) (Fig. 4A) (11, 12, 15, 16). Here, we show that the upstream helix is another conserved epitope in the S2 subunit. Sequence analyses across β-CoV lineages (Fig. 4B) showed that there are 6/21 fully conserved and 5/21 partially conserved residues in the stem helix, 8/13 fully conserved and 2/13 partially conserved residues in the fusion peptide, and 8/42 fully conserved and 11/42 partially conserved residues in the upstream helix (Fig. 4B). Here, partially conserved refers to residues in bins 6–8 and fully conserved refers to residues in bin 9 as per the consurf database scheme (23–28), where all bins 6–9 indicate slowly evolving conserved sites.

Structural analysis of postfusion structures of SARS-CoV-2 (PDB ID: 8FDW) (21), SARS-CoV (PDB ID: 6M3W) (30), and OC-43 SP S2 subunits showed that the UH region is exposed and available for binding by mAb 1871 (Fig. 2D). Furthermore, the UH region has a rmsd of 0.6 Å and 0.7 Å for SARS-CoV-2 and SARS-CoV, respectively (Fig. S8A), indicative of structural similarity and further explaining binding of mAb 1871 to all three S2 subunits.

Although mAb 1871 bound to a broad range of β-CoVs, it failed to neutralize the SARS-CoV-2 Omicron BA.1 and BA.5 VOCs (Fig. 4C; Fig. S8B). In this context, a single point mutation, N842K, occurs in the UH epitope of both Omicron BA.1 and BA.5 VOC, which we posit, based on structural modeling, may lead to steric clashes with the mAb 1871 light chain and presumably abrogate neutralization potency. This point mutation persists in more recent Omicron subvariants, which we predict would not be neutralized by 1871, considering the emerging trend of decreased activity of 1871 against viruses carrying a lysine at position 842 (Fig. 4D and E).

Like Ab S2P6 (11), which binds β-CoVs but not alphacoronaviruses, mAb 1871 also shows binding to β-CoVs (SARS-CoV, SARS-CoV-2, MERS, OC-43, and HKU-1) but does not bind to alphacoronavirus HCoV-229E (20). Molecular modeling suggests that three residues that are involved in hydrogen bonding with Lys 811, Thr 813, and Asp 839 are mutated in HCoV-229E, which could lead to changes in affinity and may explain the inability of mAb 1871 to bind to this alphacoronavirus (20) (Fig. 4D and F). Overall, our molecular understanding of the partially conserved mAb 1871 epitope in the UH aligns well with its ability to bind and neutralize sarbecoviruses.

### mAb 1871 is dependent on effector functions to confer *in vivo* protection

**Fig 5:**
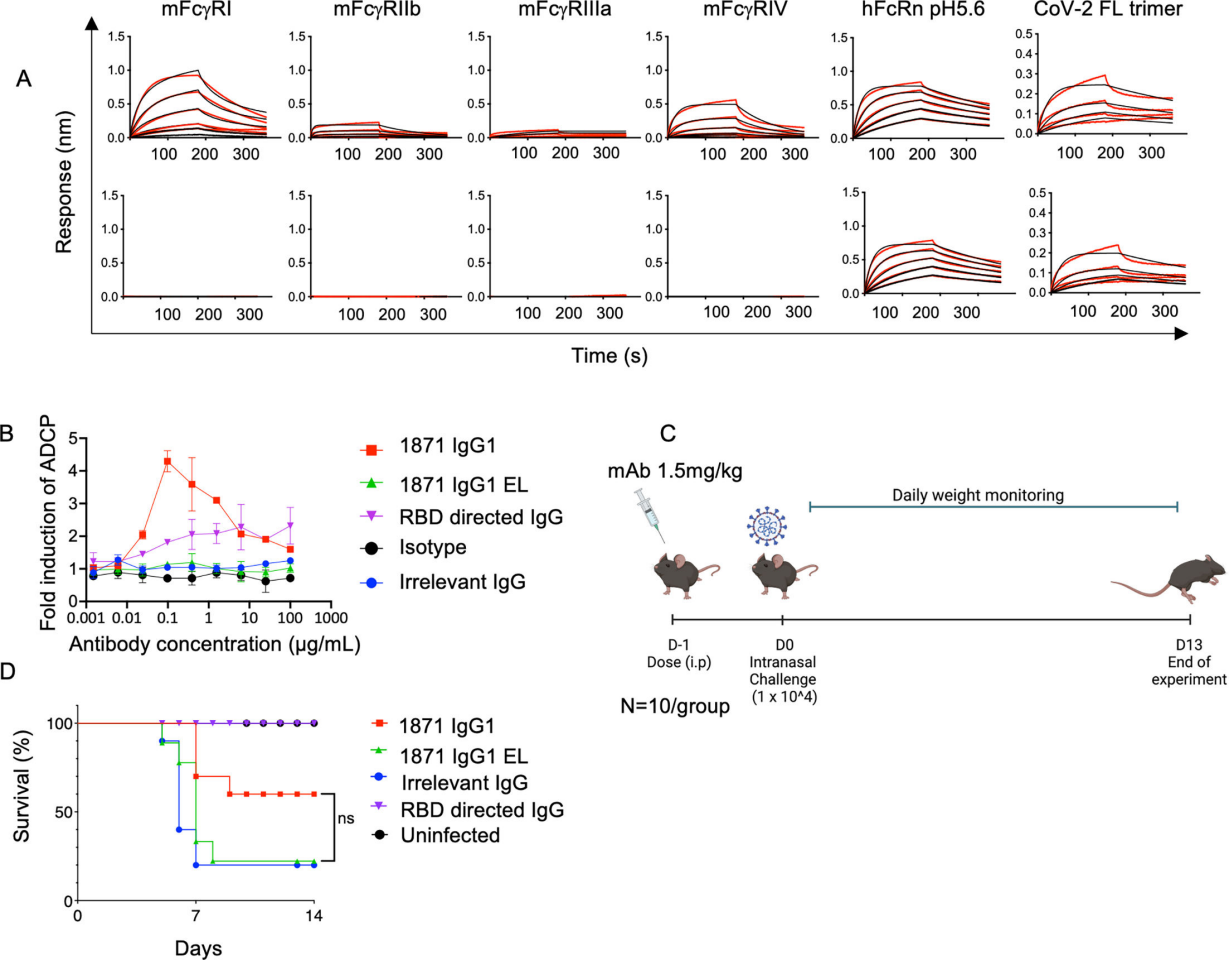
mAb 1871 in *vivo* protection is dependent on effector functions. (**A**) Sensograms of mAb 1871 IgG1 and mAb 1871 IgG1 EL binding to mouse Fc receptors FcγRI, FcγRIIb, FcγRIIIa, FcγRIV, human FcRn at pH 5.6, and SARS-CoV-2 full-length antigen. (**B**) *In vitro* Ab-dependent cellular phagocytosis, with mAb 1871 IgG1 shown in red and mAb 1871 IgG1 EL shown in green. RBD Ab control is shown in purple. Data shown are normalized as fold induction over no Ab control wells. Means from two biological replicates, each consisting of two technical replicates, are shown for each data point. (**C**) K18-hACE2 and hFcRn double-transgenic mice were dosed (1.5 mg/kg) as indicated and challenged intranasally with 1 × 10^4^ PFUs of SARS-CoV-2. Survival and weight loss (morbidity) were monitored for 14 days post-challenge. (**D**) Survival percentage monitored for 14 days post-challenge. Survival curves were compared using Mantel-Cox (*P* = 0.054) and Gehan-Breslow-Wilcoxon (*P* = 0.051) tests. The difference in survival did not reach statistical significance, though both tests strongly trend towards improved survival with the 1871 IgG1.

SP S2 subunit-directed Abs have been shown to confer *in vivo* protection despite weaker *in vitro* neutralization potency compared to RBD-directed Abs (10–12). This effect has been attributed to leveraging Fc effector functions through viral clearance and promoting antiviral immune responses (10, 11). Here, to assess the role of effector functions for the potency of mAb 1871, we generated mAb 1871 with a wild-type (WT) human Immunoglobulin 1 (IgG1) Fc domain and one with a human IgG1 Fc containing L234A, L235A, and P329G mutations to ablate binding to Fcγ receptors (herein referred to as mAb 1871 IgG1 EL). Mutations in the Fc of IgG1 EL did not affect binding to SARS-CoV-2 full-length antigen compared to the WT IgG1 and maintained the expected, unaltered high-affinity binding to human FcRn at pH 5.6 (Fig. 5A). However, mAb 1871 IgG1 EL displayed the expected, ablated binding to other mouse Fc receptors compared to mAb 1871 IgG1 (Fig. 5A). To investigate the effector function impact of this loss of Fc receptor binding, we assessed *in vitro* Ab-dependent cellular phagocytosis (ADCP) using a multiplexed bead-based assay. mAb 1871 IgG displayed high induction of ADCP, whereas, as expected, mAb 1871 IgG EL did not display any ADCP function (Fig. 5B).

To assess the impact of ablated Fc effector functions on *in vivo* protection against SARS-CoV-2, K18 human angiotensin-converting enzyme 2 (hACE2) and human FcRn double-transgenic mice were intraperitoneally treated with 1.5 mg/kg of the mAb 1871 IgG1 and mAb 1871 IgG1 EL and then challenged intranasally with a lethal dose of 1 × 10^4^ PFUs of SARS-CoV-2 USA-WA1/2020 (Fig. 5C). Despite a relatively moderate neutralization potency (IC_50_ of 1.5 µg/ml), mAb 1871 IgG1 provided 60% survival. Comparatively, mAb 1871 IgG1 EL only conferred 20% survival at the same dose, comparable to the irrelevant IgG control, indicative of a protective role conferred by Fc effector functions. The difference in survival did not reach statistical significance as assessed by both the Mantel-Cox (*P* = 0.054) and Gehan-Breslow-Wilcoxon test (*P* = 0.051), though both tests trend towards improved survival with the 1871 IgG1 (Fig. 5D; Fig. S9). These results suggest that Abs targeting the UH epitope in the SP S2 subunit rely on effector functions to provide protection against SARS-CoV-2 challenge.

## DISCUSSION

Currently, two β-CoV-based diseases, MERS and COVID-19, are on the World Health Organization’s (WHO) priority disease list (36). During the recent COVID-19 pandemic, the global toll reported reached over 7 million deaths (37). Repeated emergence of β-CoV-based epidemics caused by zoonotic transmissions warrants the development of therapeutics or prophylactics to combat future outbreaks (38). Several Abs that received emergency use authorization during the COVID-19 pandemic were revoked due to rapid mutations in their targeted epitopes, and SARS-CoV-2 vaccines are being consistently modified to cater to the new VOCs (4, 7). Therefore, the development of pan-β-CoV therapeutics and prophylactics is paramount to support readiness and global stability in the event of a future coronavirus pandemic.

The SP S2 subunit, comprising the fusion machinery, is more conserved than the SP S1 subunit, which is subjected to high immunological pressure. Hence, it is hypothesized that Abs directed against the S2 subunit may provide an opportunity to develop pan-β-CoV therapeutics. Abs directed against two conserved S2 subunit epitope bins, namely the stem helix and fusion peptide, have been described (10–12, 15, 16). In addition, a few antibodies targeting other conserved epitopes in the S2 subunit, such as the HR2 epitope recognized by murine hMab5.17 (39) and the S2 apex epitope (between HR1 and CH) recognized by mAb 54043-5 (40), have been identified. Murine hMab5.17 displayed neutralization across multiple SARS-CoV-2 VOCs and conferred *in vivo* protective efficacy in a hamster challenge model (39). Although 54034-5 is non-neutralizing, this antibody—isolated from a human convalescent donor—displayed broad β-CoV binding and provided 40% protection *in vivo* in an Fc-attenuated format at a high dose (12 mg/kg) (38). These findings suggest that antibodies directed against conserved epitopes in S2 may act through various mechanisms to provide broad protection against β-CoVs. Further characterization of such antibodies may help better understand their modes of action and support vaccine design strategies to target conserved epitopes.

Here, we have characterized functional and molecular details of mAb 1871, which has broad reactivity to β-CoVs. Using cryo-EM, we elucidated that mAb 1871 targets the upstream helix, a partially conserved epitope in the S2 subunit. The upstream helix consists of 43 amino acids and retains its tertiary structure in the prefusion and postfusion SP conformations. The epitope consists of highly conserved disulfide bonds that help the S2 subunit to retain its structural integrity during conformational changes. We identified that mAb 1871 does not bind to the prefusion-stabilized SP. Indeed, the upstream helix, as recognized by mAb 1871, is occluded in the prefusion conformation due to the presence of the S1 subunit. Presumably, mAb 1871 binds to the upstream helix epitope when it becomes exposed during an intermediate stage when the SP undergoes conformational changes and interferes with the fusion machinery’s ability to mediate membrane fusion as a mechanism to neutralize the virus.

Although we show that mAb 1871 has broad reactivity to a β-CoV panel and targets a partially conserved epitope in the S2 subunit, our study has some limitations. Indeed, this Ab loses neutralization against the SARS-CoV-2 Omicron BA.1 VOC likely due to an Asn842 to Lys842 mutation in the binding site, which presumably leads to steric clashes and loss of binding. Since this is one of the first mAbs described against the UH site, there may be further opportunities to identify other Abs that target more conserved residues in the region for improved breadth.

RBD-directed mAbs are potent neutralizers; for example, mAbs such as ADPT03019, ADPT0980, and ADPT03995 have *in vitro* neutralization potency in the range of 1–4 ng/mL (20), whereas mAb 1871, which is S2 directed, is a weak neutralizer with a potency of 1,500 ng/mL against SARS-CoV-2 US/WA-1. In a K18 hACE2 mouse model challenge study, when challenged intranasally with 1 × 10^5^ PFU of SARS-CoV-2/US WA1/2020, these RBD-directed Abs had greater than 80% survival when administered at 1.5 mg/kg and mice treated with mAb 1871 at 5 mg/kg had 70% survival and presented minimal morbidity signs (weight loss) at 10 days post-infection (20). Here, we show that K18-hACE2 hFcRn double transgenic mice treated at the lower dose of mAb 1871 (1.5 mg/kg) and challenged with a lethal dose of 1 × 10^4^ PFU of SARS-CoV-2/WA1/2020 provided 60% survival. Although we observed continued protection at a lower dose compared to the 5 mg/kg dose in the initial study, a variation in the mouse model and challenge dose between the studies is a criterion that could also play a role in the mAb circulation, exposure, and protection efficacy. Furthermore, in a similar transgenic hACE2 mouse model, S2-directed mAb CC40.8 displays dose-dependent protection (300, 100, 50, and 10 µg per animal) with minimal weight loss, and mAb ADPT01823 demonstrates 60% survival when administered at 5 mg/kg. These examples illustrate the protective efficacy of S2-directed antibodies in a transgenic hACE2 mouse model. However, it is important to note that these antibodies have not been evaluated in a double transgenic hACE2/hFcRn model, limiting direct comparison. Future benchmarking experiments of S2-directed mAbs in the same challenge model and experimental setup would enable direct activity relationships to be derived.

We hypothesized that even though mAb 1871 has weak neutralization compared to RBD-directed Abs, its *in vivo* protection might be due to Fc effector functions. To evaluate this possibility, we generated mAb 1871 IgG1 and mAb 1871 IgG1 EL with Fc silencing mutations L234A, L235A, and P329G to ablate binding to Fcγ receptor. Our results confirmed that mAb 1871 IgG1 EL exhibited reduced binding to Fc gamma receptors and a loss of ADCP activity. Interestingly, mAb 1871 IgG1 exhibited high ADCP activity at lower concentrations, but this activity diminished at higher concentrations. This effect may be attributed to the prozone effect or increased monovalent binding to Fcγ receptors, which could reduce receptor clustering and downstream signaling or potential antibody aggregation at higher concentrations. *In vivo* studies demonstrated that, when treated at the same dose, mAb 1871 IgG1 EL provided only 20% survival when compared to 60% survival with mAb 1871 IgG1. Notably, mice treated with mAb 1871 exhibited greater initial weight loss followed by recovery, whereas the group treated with RBD-directed antibody showed minimal weight loss. This may be attributed to the difference in the mechanism of action of these antibodies, and that mAb 1871 may rely more heavily on Fc effector function than on neutralization, which may allow some early viral replication. This result underscores the importance of Fc effector function for this S2 subunit-directed mAb. Our data contribute to expanding evidence that SP S2-subunit-directed mAbs benefit from effector function.

Other examples include CC40.8 (10), 76E1 (13), CC99.103 (12), CC25.106 (12), CC95.108 (12), and CC68.109 (12) that show improved *in vivo* protection despite relatively low neutralization potency compared to RBD-directed Abs. Fc effector functions have also been reported to be significant in therapeutic settings when compared to prophylactic settings for RBD-directed Abs (41). In prophylactic studies in mouse models, Fc-silenced RBD-directed Abs have shown mixed results. COV2-2050 (42) failed to prevent mouse weight loss or reduce viral load, CV3-1 (43) showed a 100% mortality rate, SC31 (44) had 50% worse survival rates, and WRAIR-2123 (45) had 70% survival. These findings emphasize the critical role of neutralization in providing protection, especially in the absence of effector functions for RBD-directed Abs. Further studies could explore Fc engineering strategies to enhance effector functions, potentially improving the protective efficacy of SP S2 subunit-directed Abs. Additionally, combining RBD-directed and S2-directed antibodies in antibody cocktails or multispecific formats (46) could enhance both potency and breadth of neutralization. Identifying antibodies targeting distinct S2 epitopes may facilitate such approaches, contributing to the development of more effective pan-β-CoVs therapeutic strategies.

Here, we elucidate the binding of mAb 1871 to the upstream helix in the postfusion state, providing a glimpse into this conformation recognized by a mAb recovered from natural infection. Further studies to understand the events that lead to the unmasking of this epitope could provide detailed insight into the mechanism of fusion inhibition. The epitope uncovered for mAb 1871 also provides molecular details to be leveraged for structure-guided vaccine design efforts that specifically target the SP S2 subunit.

## MATERIALS AND METHODS

### Expression and purification of Fab and IgG

Genes encoding the heavy and light chains of Fab and IgG were synthesized and cloned by Geneart (Life Technologies) into a pcDNA3.4 expression vector. Fab and IgG were transiently expressed in HEK-293F cells (Thermo Fisher Scientific). Cells were seeded at a density of 0.8 × 10^6^ cells/mL and were incubated for 24 h at 37°C, 8% CO_2_ at 125 rpm in a Multitron Pro Shaker (InforS HT). Within 24 h, cells were co-transfected with 90 µg of DNA consisting of heavy chain and light chain in a 2:1 ratio, preincubated with polyethylenimine (PEI) (Polysciences) at room temperature for 10 min. After 6–7 days, cell suspensions were harvested by spinning at 6,000 rpm for 20 min, and supernatants were filtered using a 0.2 µm Steritop filter (EMD Millipore). Fab was purified using KappaSelect affinity (Cytiva), and IgGs were purified using protein A affinity chromatography (Cytiva); eluted using 100 mM glycine (pH 2.2), and neutralized with 1 M Tris-HCl (pH 9.0). Fab fractions were further purified using cation exchange chromatography (MonoS, Cytiva), and IgG fractions were purified using size exclusion chromatography (Superdex200, Cytiva). All IgGs for *in vivo* experiments were tested to ensure endotoxin concentrations were below 3.5 EU/mL at 1 mg/mL concentration.

### Design, expression, and purification of antigens for biolayer interferometry

Gene encoding the OC43 antigen, consisting of the extracellular S2 subunit domain (753-1240 GenBank: UOP57224.1) with a downstream TEV protease cleavage site, T4 foldon domain, and Histidine tag, was synthesized and cloned by Geneart (Life Technologies) into a pcDNA3.4 expression vector. HEK-293S (GnT I^-/-^; Thermo Fisher Scientific) cells (200 mL) were seeded at a density of 0.8 × 10^6^ cells/mL and were incubated for 24 h at 37°C, 8% CO_2_ at 125 rpm in a Multitron Pro Shaker (InforS HT). Cells were transfected with 50 µg of DNA preincubated with polyethylenimine (PEI) (Polysciences) at room temperature for 10 min. After 6–7 days, cell suspensions were harvested by spinning at 6,000 rpm for 20 min, and supernatants were filtered using a 0.2 µm Steritop filter (EMD Millipore). OC43 S2 subunit was purified using HisTrap affinity chromatography column (Cytiva) followed by size exclusion chromatography (Superose 6, Cytiva). Full-length prefusion stabilized SARS-CoV-2 spike ectodomain (BEI NR52394) was purified as described (47) using HisTrap-NiNTA column (Cytiva) followed by a Superose 6 column (Cytiva) in a 20 mM phosphate pH 8.0, 150 mM NaCl buffer. Recombinant mouse Fc receptors (FcγRI, FcγRIIb, FcγRIIIa, and FcγRIV) and human FcRn were expressed and purified as described (34, 48) using a HisTrap-NiNTA (Cytiva) column followed by Superdex 200 increase column (Cytiva).

### Biolayer interferometry

An Octet Red96 Biolayer Interferometer instrument (Sartorius ForteBio) was used to assess binding kinetics. His-tagged full-length spike proteins SARS-CoV (Sino Biological, Cat#40634-V08B), SARS-CoV-2, MERS (Sino Biological, Cat#40069-V08B), HKU-1 (Sino Biological, Cat#40606-V08B), OC-43 (Sino Biological, Cat#40607-V08B), HCoV-229E (Sino Biological, Cat#40605-V08B) or S2 subunit SARS-CoV (Sino Biological, Cat#40150-V08B), SARS-CoV-2 (Sino Biological, Cat#40590-V08B), MERS (Sino Biological, Cat#40070-V08B), OC-43 or SARS-CoV-2 full-length prefusion stabilized trimer was loaded onto Ni-NTA biosensors (Sartorius ForteBio) until it achieved a 0.5–0.8 nm signal response or reached a maximum loading time of 800 s. The antigen loading time varied from 50 s to 800 s, resulting in 0.5–0.8 nm signal response depending on antigen (Fig. S1^0^). Association was measured by transferring sensors to wells containing serial dilutions of the IgGs or Fab (500, 250, 125, 62.5, 31.2, and 15.6 nM) for 180 s, and dissociation rates were measured by transferring the sensors to buffer-containing wells for 180 s. Statistics are derived from concentrations across serial dilutions calculated from Sartorius Fortebio software. His-tagged mouse FcγRI, FcγRIIb, FcγRIIIa, FcγRIV, or human FcRn were used to assess the binding of the antibody to Fc receptors. The following biotinylated peptides of the upstream helix were synthesized at GenScript: 12aa–SSPKVTIDCAAFGSGSG, 32aa–SSPKVTIDCAAFVCGDYAA CKLQLVEYGSFCDGSGSG, and 43aa–SPKVTIDCAAFVCGDYAACKLQLVEYGSFCDNINAILT EVNEGSGSG. Peptides were loaded on SAX biosensors (Sartorius ForteBio) until a 0.8 nm signal response was reached, and association and dissociation were measured as described for Ni-NTA sensors.

### Crystallization and structure determination of 1871 Fab

Purified 1871 Fab was concentrated to 5 mg/mL, and crystallization trials were set up by sitting drop vapor diffusion using the MCSG1 sparse screen matrix in a 1:1 protein:reservoir ratio. Crystals grew in a condition containing 0.1 M sodium acetate, pH 4.6, and 3.5 M sodium formate. Crystals were cryo-protected in 15% (vol/vol) ethylene glycol and flash frozen. X-ray diffraction data were collected at the Canadian Light Source on beamline (CMCF-ID). The data set was processed using XDS (49) and XPREP (50). Phaser (51) was used to determine phases with a 1871 Fab search model predicted by Abodybuilder (52). Further refinement was performed using Phenix (53), and structure building was done using Coot (54). All software was accessed through SBGrid (55). Pymol (56) was used to generate figures. The structure has been deposited in the Protein Data Bank under the accession number PDB ID 9NQ3.

### Expression and purification of OC43 S2 construct for antibody-antigen structure determination

OC43 S2 extracellular domain was purified as described above. For structure determination, the fractions collected from HisTrap affinity chromatography were further processed. Fractions collected were then treated with Endoglycosidase H (New England Biolabs) for 30 min at 4°C and purified via size exclusion chromatography (Superose6, Cytiva).

### Cryo-EM structure determination of the OC43 S2-1871 Fab co-complex

A complex of the OC43 S2 and 1871 Fab was obtained by mixing excess Fab:antigen to ensure full occupancy of the binding sites. After 30 min of incubation at 4°C, the complex was purified using size exclusion chromatography (Superose6, Cytiva). The fractions of interest were then concentrated to 250 µg/mL. Two gold grids made in-house were prepared as previously described (57) and were glow-discharged for 15 s at a current of 25 mA. Using a Leica EM GP2 plunge freezer, two grids were prepared using 3 s preblot time, 3 s blot time, and 3 µL of sample volume.

A Titan Krios equipped with a Falcon 4 camera was used to collect a total of 3,325 and 1,470 movies with 0° and 40° tilt, respectively (54 e^-^/Å^2^, 1.03 Å/pix). All single particle analysis was performed using CryoSPARC v4.3.1 (58). Patch motion correction, patch CTF estimation, exposure curation, blob picking, template picking, and particle stack cleaning using multiple 2D classifications (and the rebalance classes job for the 0° tilt data set) were done separately for the two datasets, with a total of 324,859 and 119,216 particles Fourier-cropped by a factor of 2 for the 0° and 40° tilt data sets, respectively. Combining the two particle stacks and running 2D classification to remove junk particles resulted in a total of 444,075 particles. Ten thousand particles from the combined particle stack were used for *ab initio* reconstruction. Templates were created from the resulting map for a second template picking from the 40° tilt micrographs, to maximize the number of rare views. The resulting particle stack and the 0° tilt particle stack prior to 2D classification with the original 40° tilt particles underwent exposure curation to remove low-quality particles by exclusion of low-quality micrographs; a combined total of 367,895 particles was kept. This particle stack and the map from ab initio reconstruction were used as input for non-uniform (NU) refinement with C1 symmetry, reaching a resolution of 4.48 Å. The 3D classification with three classes was performed using a mask that excluded density from the conserved domain of the 1871 Fab particles, followed by heterogeneous refinement with C3 symmetry. One class (109,888 particles, 4.41 Å) contained most of the density for OC43 and was used for three rounds of NU-refinement with alternating symmetry settings (C1, C3, C1) to aid in correct particle angular assignment; the resulting map reached 4.48 Å. The particle stack was re-extracted without Fourier cropping and used for NU-refinement with C1 symmetry, reaching a resolution of 3.09 Å with 109,156 particles. The particles were split into ten exposure groups, underwent local and global CTF refinement (trefoil and tilt only), NU-refinement (C1 symmetry), then another round of local and global CTF refinement (all corrections including magnification anisotropy), and two final rounds of NU-refinement (C1 symmetry, then C3 symmetry); this map (map A) reached a final resolution of 2.60 Å. To resolve more of the density present in the triple helix bundle, three NU-refinements were performed on map A: C3 symmetry refinement, C3 symmetry with a mask excluding the triple helix bundle (to ensure the angular assignment of the particles is correct), and C3 symmetry with a mask excluding the 1871 Fab particles; this map (map B) reached a resolution of 2.74 Å. The crystal structure of 1871 Fab and OC43 S2 subunit model obtained from Alphafold-2 (59) was manually docked into the cryo-EM map using UCSF Chimera (60). Manual adjustment based on this model was done using COOT (54), followed by iterative rounds of refinement using COOT (54) and Phenix (53). Interface residues were identified using PDB PISA (61). Figures were made using UCSF Chimera (60) and Pymol (56). The structure has been deposited in the Protein Data Bank under the accession number PDB ID 9NQZ and the Electron Microscopy Data Bank under the accession number EMD-49708. AF3 modeling (33) was performed with the SARS-CoV-2 spike sequence (Uniprot P0DTC2) and 1871 Fab.

### Pseudovirus neutralization

SARS-CoV-2 pseudotyped viruses (PsV) were generated through transient transfection of 293T cells (ATCC) with lenti-viral backbone (BEI NR52516), structural and regulatory genes (BEI NR52518, NR52517, NR52519), and SARS-CoV-2 spike of interest (SARS-CoV-2/WIV04/2019 wildtype (BEI NR52516), Omicron/BA.1 (Scripps Research) (34, 47) or Omicron/BA.5. Gene encoding Omicron BA.5 spike protein was synthesized and cloned by GeneArt (Thermo Fisher Scientific) in pcDNA3.4 backbone. Neutralization was assessed by single-cycle neutralization assays using 293-ACE2 cells (BEI NR52511), as previously described (34). Two biological replicates with two technical replicates were performed for each molecule.

### Antibody-dependent cellular phagocytosis (ADCP)

ADCP was measured using the FcγRIIa-H ADCP Bioassay kit (Promega), with CHO-K1/Spike stable cell line (GenScript) as the target cells. An assay was performed according to the manufacturer’s protocol. In brief, 6,000 target cells in 25 µL of assay buffer were plated into each well of a white, clear-bottom flat 96-well plate, and 25 µL of diluted antibody at 2X final concentration was added. After incubating target cells and antibody at 37°C for 15 min, 30,000 of freshly thawed FcγRIIa-H Effector Cells in 25 µL were added to each well for an E:T ratio of 5:1, and the plate was incubated for 6 h at 37°C. To read out the plate, 75 µL of prepared Bio-Glo Reagent was added to each well and incubated at room temperature for at least 5 min. Luminescence was measured on a plate reader (BioTEK). Data were normalized and then plotted in GraphPad Prism (GraphPad Software, Inc.).

### Mice

Ten- to 13-week-old, K18 human ACE2 and human FcRn double-transgenic mice (B6.Cg-Tg(K18-ACE2)2Prlmn Tg(FCGRT)32Dcr Fcgrttm1Dcr/2J, Jackson Labs, Cat#037043) with an average body weight of 25 g were used in this study.

### Mice and SARS-CoV-2 challenges

The protective efficacy of mAb 1871 IgG and mAb 1871 IgG EL was tested in female and male, 10- to 13-week-old mice (*n* = 10/Ab avg wt. 25 g) in human FcRn double-transgenic mice (B6. Cg-Tg(K18-ACE2)2Prlmn Tg(FCGRT)32Dcr *Fcgrt^tm1Dcr^*/2J; The Jackson Labs, Cat. No. 037043). The mice were first given the indicated mAb at 1.5 mg/kg. At 24 h after mAb delivery, mice were challenged intranasally (25 µL/nostril) with 1.0 × 10^4^ PFU SARS-CoV-2/human/USA/WA-CDC-WA-1 (GenBank MN985325) at passage 6. Next-generation sequencing was used to confirm that the virus stocks were 100% identical to the original BEI Resources P4 stock as described (62). Working stocks were also confirmed to lack the Bristol deletion and other deletions/mutations (62). Mice were observed and weighed daily over a 14-day experimental period. Any animal that lost ≥20% body weight was humanely euthanized. Survival and morbidity were assessed.

### Quantification and statistical analysis

All data and statistical analyses were performed using Prism v9.3.1 (GraphPad Software Inc). Survival curves were compared using Mantel-Cox and Gehan-Breslow-Wilcoxon tests.

## Data Availability

The cryo-EM and crystal structures have been deposited to the Electron Microscopy Data Bank (EMD-49708) and the Protein Data Bank (PDB IDs 9NQZ and 9NQ3).

## SUPPLEMENTAL MATERIAL

Supplemental figures jvi.01523-25-s0001.pdf Figures S1 to S10.

Supplemental tables jvi.01523-25-s0002.docx Tables S1 to S3.

ASM does not own the copyrights to Supplemental Material that may be linked to, or accessed through, an article. The authors have granted ASM a non-exclusive, world-wide license to publish the Supplemental Material files. Please contact the corresponding author directly for reuse.
