# Supplementary material for: Human antibody targeting of coronavirus spike S2 subunit is associated with protection mediated by Fc effector functions
Source: J Virol. 2025 Nov 12;99(12):e01523-25. doi: 10.1128/jvi.01523-25 (PMC12724371; doi:10.1128/jvi.01523-25)
Supplement: Supplemental figures — Figures S1 to S10. [file jvi.01523-25-s0001.pdf]

Figure S1

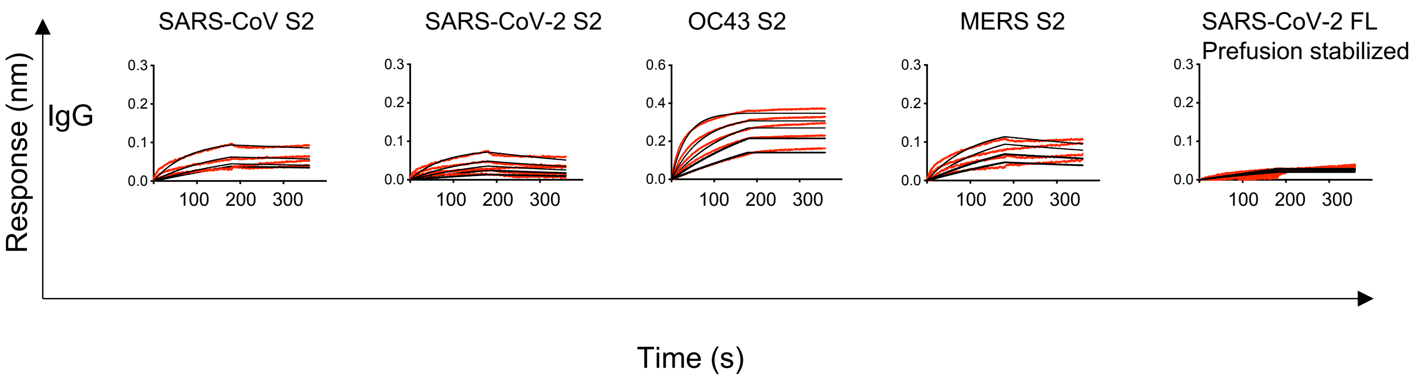

SARS-CoV S2
SARS-CoV-2 S2
OC43 S2
MERS S2
SARS-CoV-2 FL
Prefusion stabilized
Response (nm)
IgG
Time (s)

Figure S1. Binding profile of mAb 1871 to spike proteins. Sensograms showing binding of mAb 1871 IgG to SARS-CoV, SARS-CoV-2, OC-43 and MERS S2 subunit and no binding to SARS-CoV-2 full length prefusion stabilized trimer. Red lines represent raw data, and black lines represent global fit.

Figure S2

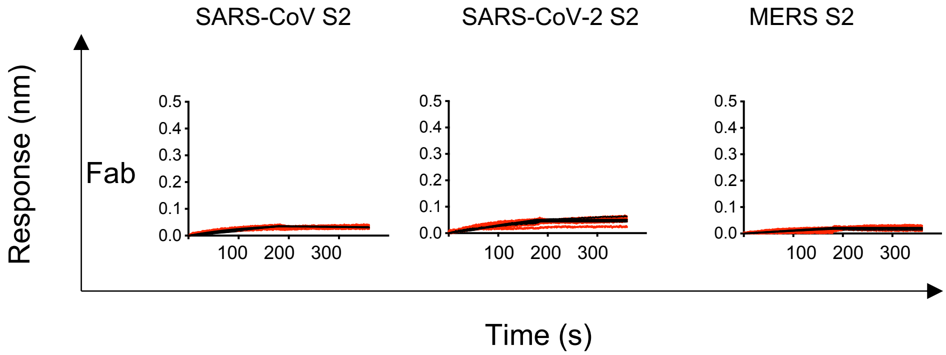

SARS-CoV S2
SARS-CoV-2 S2
MERS S2
Response (nm)
Fab
0.5
0.4
0.3
0.2
0.1
0.0
100
200
300
Time (s)

Figure S2. Binding profile of 1871 Fab to spike S2 subunit proteins. Sensograms showing binding of 1871 Fab to SARS-CoV, SARS-CoV-2, and MERS S2 subunit. Red lines represent raw data, and black lines represent global fit.

Figure S3

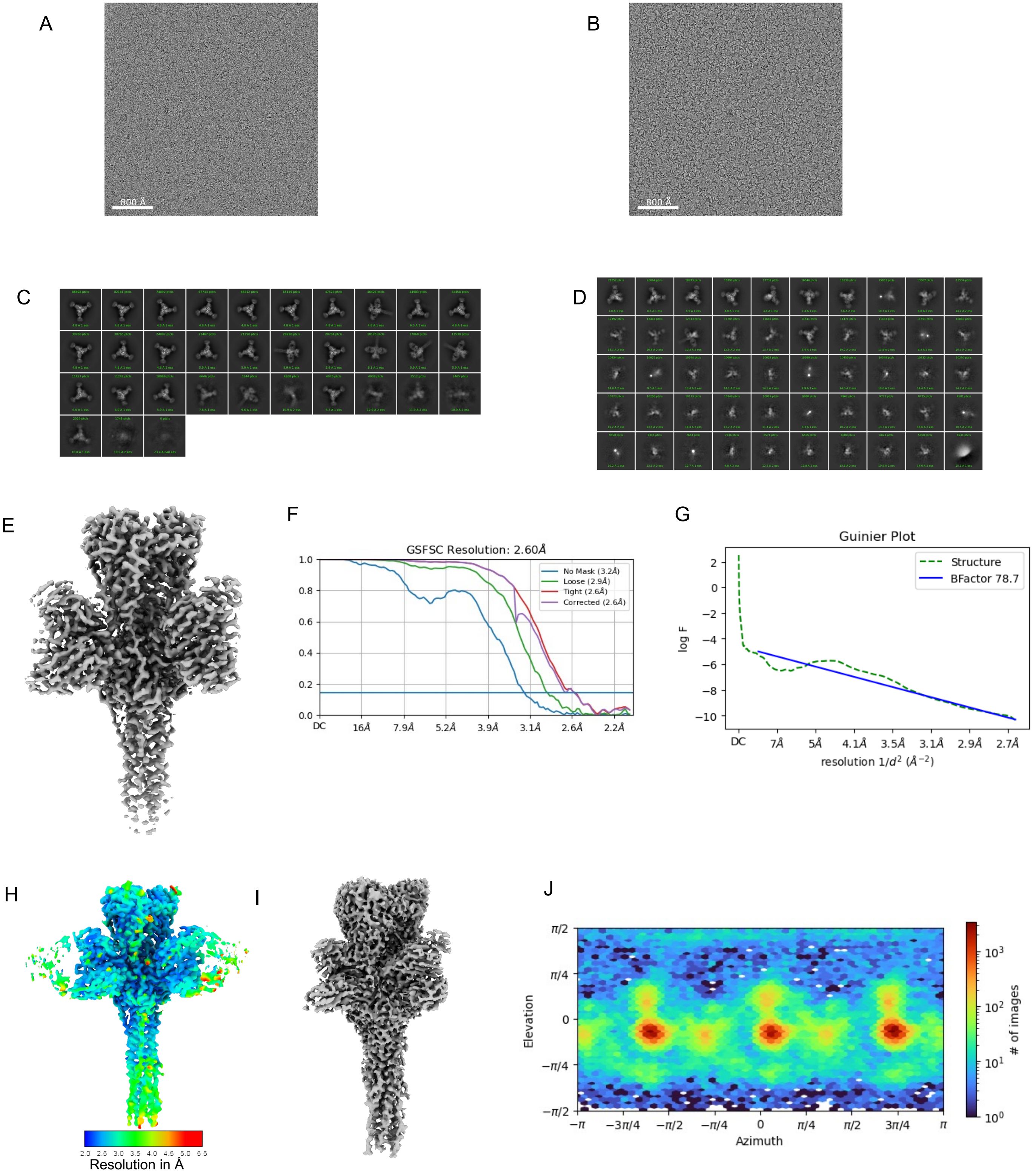
A
800 Å
B
800 Å
C
D
E
F
GSFSC Resolution: 2.60Å
No Mask (3.2Å)
Loose (2.9Å)
Tight (2.6Å)
Corrected (2.6Å)
1.0
0.8
0.6
0.4
0.2
0.0
DC 16Å 7.9Å 5.2Å 3.9Å 3.1Å 2.6Å 2.2Å
G
Guinier Plot
Structure
BFactor 78.7
log F
2
0
−2
−4
−6
−8
−10
DC 7Å 5Å 4.1Å 3.5Å 3.1Å 2.9Å 2.7Å
resolution 1/d² (Å⁻²)
H
2.0 2.5 3.0 3.5 4.0 4.5 5.0 5.5
Resolution in Å
I
J
Elevation
π/2
π/4
0
−π/4
−π/2
−π −3π/4 −π/2 −π/4 0 π/4 π/2 3π/4 π
Azimuth
# of images
10³
10²
10¹
10⁰

Figure S3. Raw electron micrographs of 1871-OC43 and resolution estimates. (A) Representative electron micrograph for the untilted dataset. **(B)** Representative electron micrograph for the 40-degree tilted dataset. **(C-D)** 2D classifications of untilted and tilted datasets. **(E)** Map of the particle from final refinement job (complete job). **(F-G)** Fourier shell Correlation for the half maps and the Guinier plot with the estimated B factor for the final refinement job of the complete complex map. **(H)** Local resolution map of complex, shades indicate different resolutions in Å. **(I)** Map of the particle for the final refinement job (stem of OC43 trimer region). (**J**) Distribution plot for untilted dataset.

Figure S4

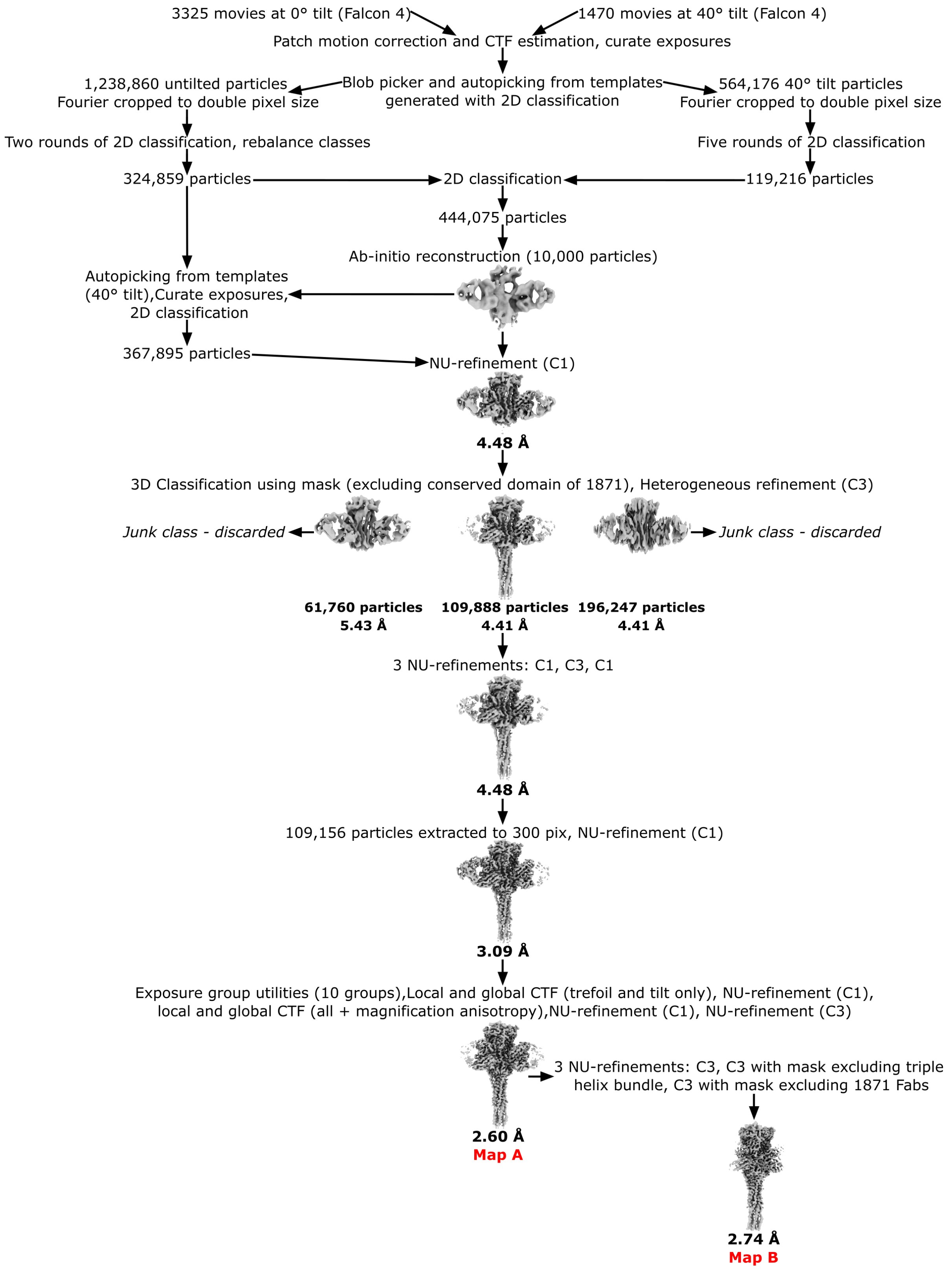
3325 movies at 0° tilt (Falcon 4)
1470 movies at 40° tilt (Falcon 4)
Patch motion correction and CTF estimation, curate exposures
1,238,860 untilted particles
Fourier cropped to double pixel size
Blob picker and autopicking from templates
generated with 2D classification
564,176 40° tilt particles
Fourier cropped to double pixel size
Two rounds of 2D classification, rebalance classes
Five rounds of 2D classification
324,859 particles
2D classification
119,216 particles
444,075 particles
Ab-initio reconstruction (10,000 particles)
Autopicking from templates
(40° tilt),Curate exposures,
2D classification
367,895 particles
NU-refinement (C1)
4.48 Å
3D Classification using mask (excluding conserved domain of 1871), Heterogeneous refinement (C3)
Junk class - discarded
Junk class - discarded
61,760 particles
5.43 Å
109,888 particles
4.41 Å
196,247 particles
4.41 Å
3 NU-refinements: C1, C3, C1
4.48 Å
109,156 particles extracted to 300 pix, NU-refinement (C1)
3.09 Å
Exposure group utilities (10 groups),Local and global CTF (trefoil and tilt only), NU-refinement (C1),
local and global CTF (all + magnification anisotropy),NU-refinement (C1), NU-refinement (C3)
3 NU-refinements: C3, C3 with mask excluding triple
helix bundle, C3 with mask excluding 1871 Fabs
2.60 Å
Map A
2.74 Å
Map B

Figure S4. Detailed Cryo-EM data processing steps in cryoSPARCv4.3.1.

Figure S5

```
OC43            SAIEDLLFDKVKLSDVGFVEAYNNCTGGAEIRDLICVQSYKGIKVLPPLLSENQISGYTL    60
SARS-CoV-2      SFIEDLLFNKVTLADAGFIKQYGDCLGDIAARDLICAQKFNGLTVLPPLLTDEMIAQYTS    60
SARS-CoV        SFIEDLLFNKVTLADAGFMKQYGECLGDINARDLICAQKFNGLTVLPPLLTDDMIAAYTA    60
                * ******:**.*:*.**:: *.:* *.   *****.*.::*:.******::: *: **

OC43            AATSASLFPPWTAAAG----VPFYLNVQYRINGLGVTMDVLSQNQKLIANAFNNALHAIQ    116
SARS-CoV-2      ALLAGTITSGWTFGAGAALQIPFAMQMAYRFNGIGVTQNVLYENQKLIANQFNSAIGKIQ    120
SARS-CoV        ALVSGTATAGWTFGAGAALQIPFAMQMAYRFNGIGVTQNVLYENQKQIANQFNKAISQIQ    120
                *  :.:    ** .**    :** ::: **:**:*** :** :*** *** **.*:  **

OC43            QGFDATNSALVKIQAVVNANAEALNNLLQQLSNRFGAISASLQEILSRLDALEAEAQIDR    176
SARS-CoV-2      DSLSSTASALGKLQDVVNQNAQALNTLVKQLSSNFGAISSVLNDILSRLDKVEAEVQIDR    180
SARS-CoV        ESLTTTSTALGKLQDVVNQNAQALNTLVKQLSSNFGAISSVLNDILSRLDKVEAEVQIDR    180
                :.: :* :** *:* *** **:***.*::***..*****: *::****** :***.****

OC43            LINGRLTALNAYVSQQLSDSTLVKFSAAQAMEKVNECVKSQSSRINFCGNGNHIISLVQN    236
SARS-CoV-2      LITGRLQSLQTYVTQQLIRAAEIRASANLAATKMSECVLGQSKRVDFCGKGYHLMSFPQS    240
SARS-CoV        LITGRLQSLQTYVTQQLIRAAEIRASANLAATKMSECVLGQSKRVDFCGKGYHLMSFPQA    240
                **.*** :*::**:***  :: :: **  *  *:.*** .**.*::***:* *::*: *

OC43            APYGLYFIHFNYVPTKYVTAKVSPGLCIAGNRGIAPKSGYFVNVNNTWMYTGSGYYYPEP    296
SARS-CoV-2      APHGVVFLHVTYVPAQEKNFTTAPAICHDGKA-HFPREGVFVSNGTHWFVTQRNFYEPQI    299
SARS-CoV        APHGVVFLHVTYVPSQERNFTTAPAICHEGKA-YFPREGVFVFNGTSWFITQRNFFSPQI    299
                **:*: *:*..***::  . ..:*.:*  *:    *:.* **  .. *: *  .:: *:

OC43            ITENNVVVMSTCAVNYTKAPYVMLNTSIPNLPDFKEELDQWFKNQTSVAPDLS-LDYINV    355
SARS-CoV-2      ITTDNTFVSGNCDVVIGIVNNTVYDPLQPELDSFKEELDKYFKNHTSPDVDLGDISGINA    359
SARS-CoV        ITTDNTFVSGNCDVVIGIINNTVYDPLQPELDSFKEELDKYFKNHTSPDVDLGDISGINA    359
                ** :*..* ..* *       .: :   *:* .******::***:**   **. :. **.

OC43            TFLDLQVEMNRLQEAIKVLNHSYINK-DIGTYEYGS-------------------------    390
SARS-CoV-2      SVVNIQKEIDRLNEVAKNLNESLIDLQELGKYEQYIKWPWYIWLGFIAGLIAIVMVTIML    419
SARS-CoV        SVVNIQKEIDRLNEVAKNLNESLIDLQELGKYEQYIKWPWYVWLGFIAGLIAIVMVTILL    419
                :.:::* *::**:*. * **.* *:  ::*.**

OC43            --------------------------------------- 390
SARS-CoV-2      CCMTSCCSCLKGCCSCGSCCKFDEDDSEPVLKGVKLHYT 458
SARS-CoV        CCMTSCCSCLKGACSCGSCCKFDEDDSEPVLKGVKLHYT 458
```

Figure S5. Sequence alignment of S2 subunit of OC43, SARS-CoV and SARS-COV-2

Figure S6

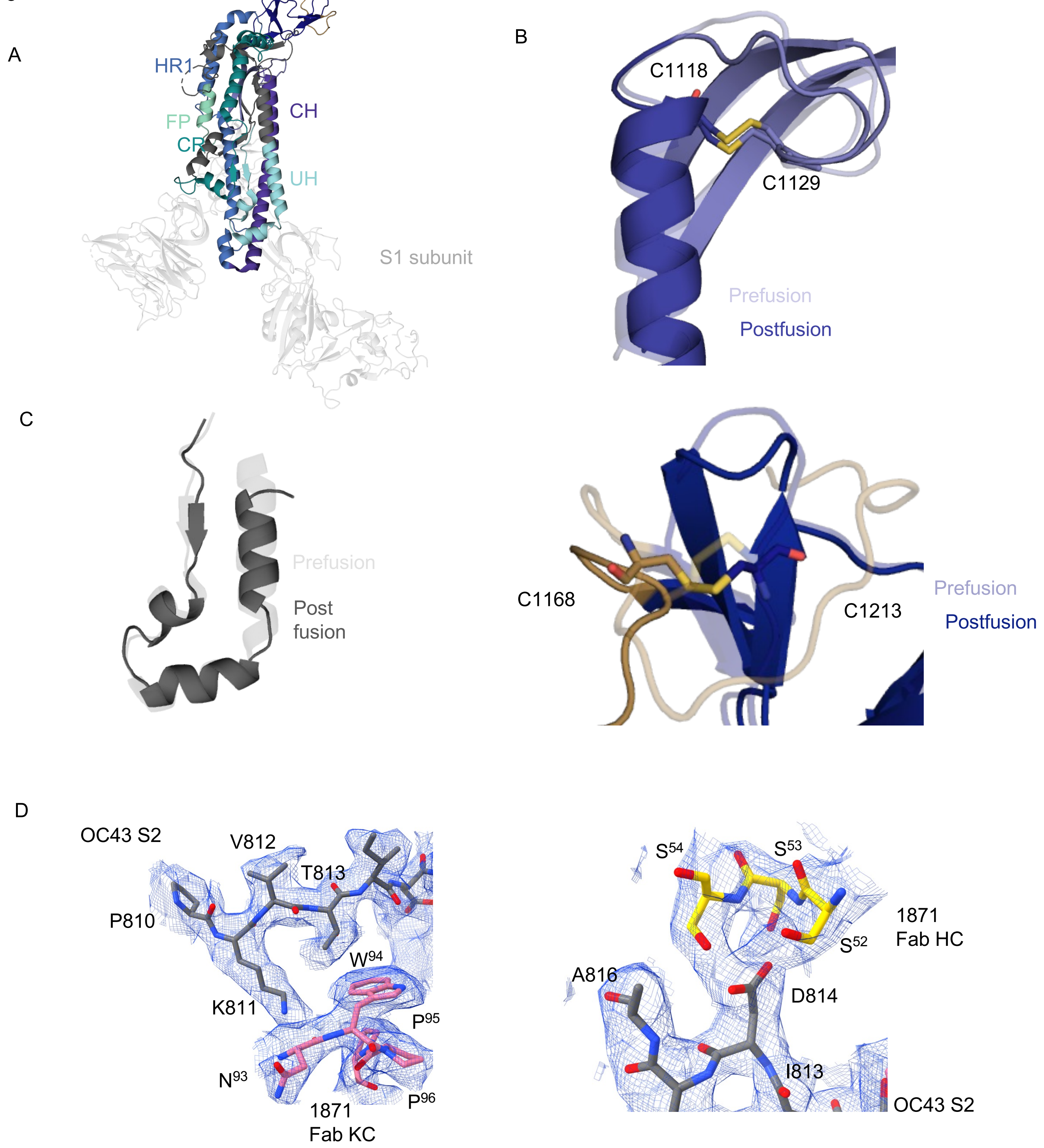

A
HR1
FP
CR
CH
UH
S1 subunit
B
C1118
C1129
Prefusion
Postfusion
C
Prefusion
Post
fusion
C1168
C1213
Prefusion
Postfusion
D
OC43 S2
V812
T813
P810
W94
K811
P95
N93
1871
Fab KC
P96
S54
S53
1871
Fab HC
S52
A816
D814
I813
OC43 S2

Figure S6. Comparison of OC-43 spike prefusion and postfusion structures. (A) Different domains are colored in the prefusion OC-43 spike (PDB ID: 7PNM)(25). **(B)** Comparison of disulphide bonds in prefusion and postfusion structures. Residues involved in disulphide bond are shown as sticks and disulphide bond is shown in yellow. **(C)** Comparison of OC-43 UH in prefusion shown in transparent grey and post fusion shown in dark grey. (**D**) Model of OC43 S2 and atomic model of Fab 1871 (PDB ID: 8DNN) fit into cryoEM map.

Figure S7

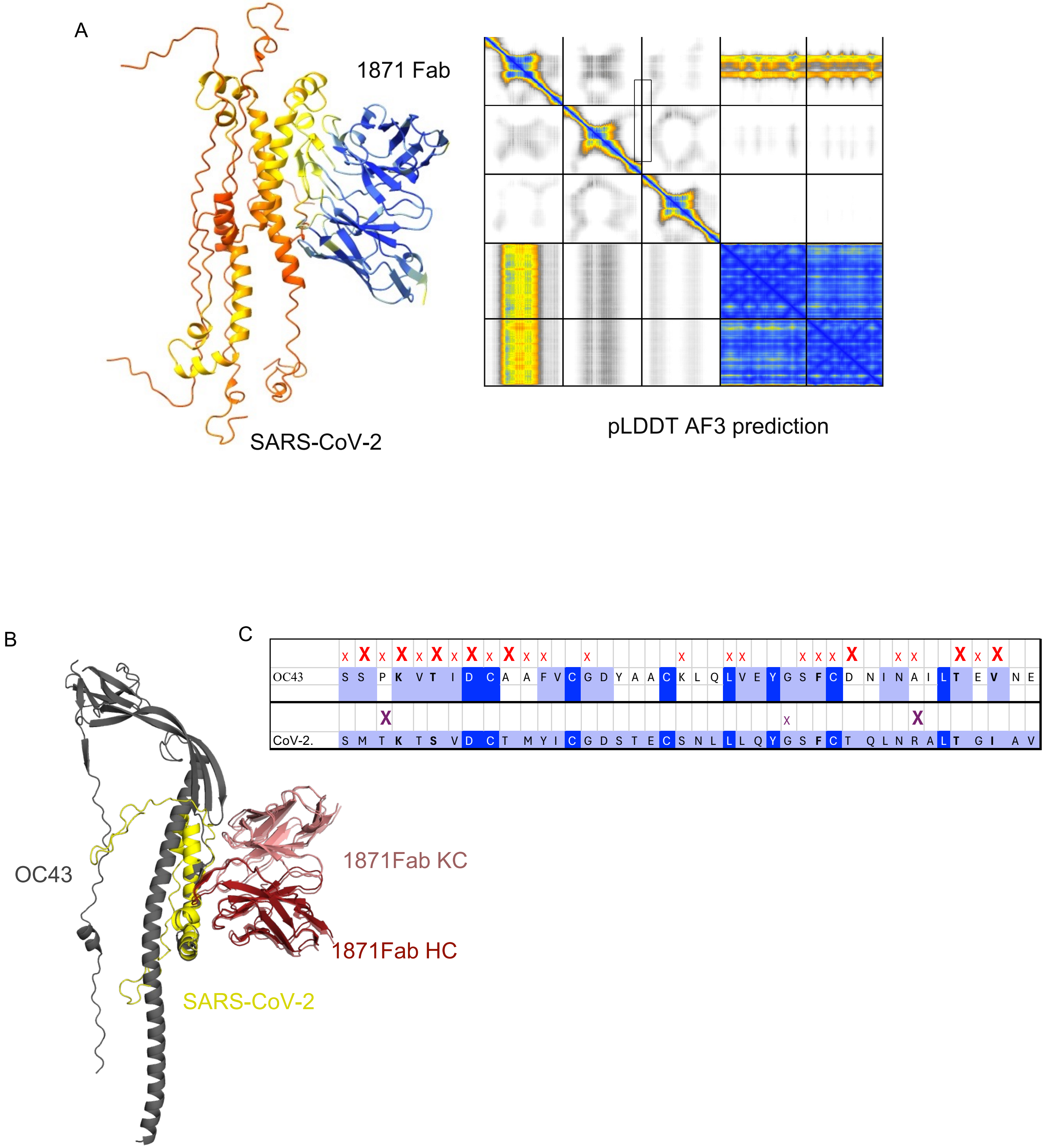
A
1871 Fab
SARS-CoV-2
pLDDT AF3 prediction
B
OC43
1871Fab KC
1871Fab HC
SARS-CoV-2
C
x X x X x X x X x X x x x x x x x x x X x x X x X
OC43 S S P K V T I D C A A F V C G D Y A A C K L Q L V E Y G S F C D N I N A I L T E V N E
X x X
CoV-2. S M T K T S V D C T M Y I C G D S T E C S N L L L Q Y G S F C T Q L N R A L T G I A V

Figure S7. Alphafold model of SARS-CoV-2 UH interaction with 1871 Fab. (A) AF3 model of SARS-CoV-2 UH with 1871 Fab. The pLDDT confidence map shows the atomic confidence metric - dark blue (>90), light blue (70-90), yellow/orange (50-70) and red (<50). **(B)** Alignment of a single protomer of AF3 SARS-CoV-2 model with 1871 Fab and OC-43 upstream helix with 1871 Fab. **(C)** Sequence conservation in UH epitope of OC43 and SARS-CoV-2. Sequences colored based on BLOSUM62 score of percentage identity. Red X's indicate interface residues and bold red X's indicate residues involved in hydrogen bonding. Purple X's indicate additional predicted interface residues.

Figure S8

A

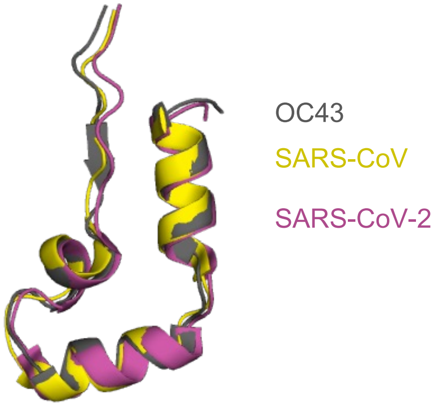
OC43
SARS-CoV
SARS-CoV-2

B

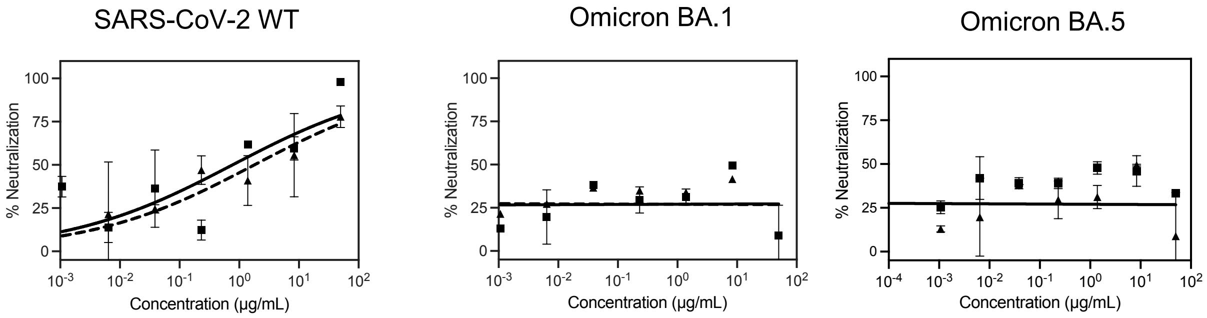
SARS-CoV-2 WT
Omicron BA.1
Omicron BA.5
% Neutralization
100
75
50
25
0
Concentration (µg/mL)
10^{-4}
10^{-3}
10^{-2}
10^{-1}
10^{0}
10^{1}
10^{2}

Figure S8. Comparison of UH in β-CoVs. (A) Comparison of UH conformation between postfusion structures of OC-43 shown in grey, SARS-CoV(23) (PDB ID: 6M3W) colored in yellow and SARS-CoV-2(22) (PDB ID: 8FDW) colored in pink. **(B)** SARS-CoV-2 PsV neutralization potency of 1871 IgG. The mean ± SD for two technical replicates are shown in each plot. The means ± SD for two biological replicates consisting of two technical replicates each are shown in each plot.

Figure S9

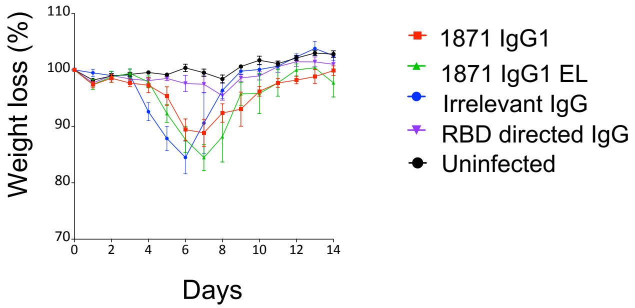
Weight loss (%)
110
100
90
80
70
0
2
4
6
8
10
12
14
Days
1871 IgG1
1871 IgG1 EL
Irrelevant IgG
RBD directed IgG
Uninfected

Figure S9. Mouse morbidity. Percent weight loss in experimental and control groups.

Figure S10

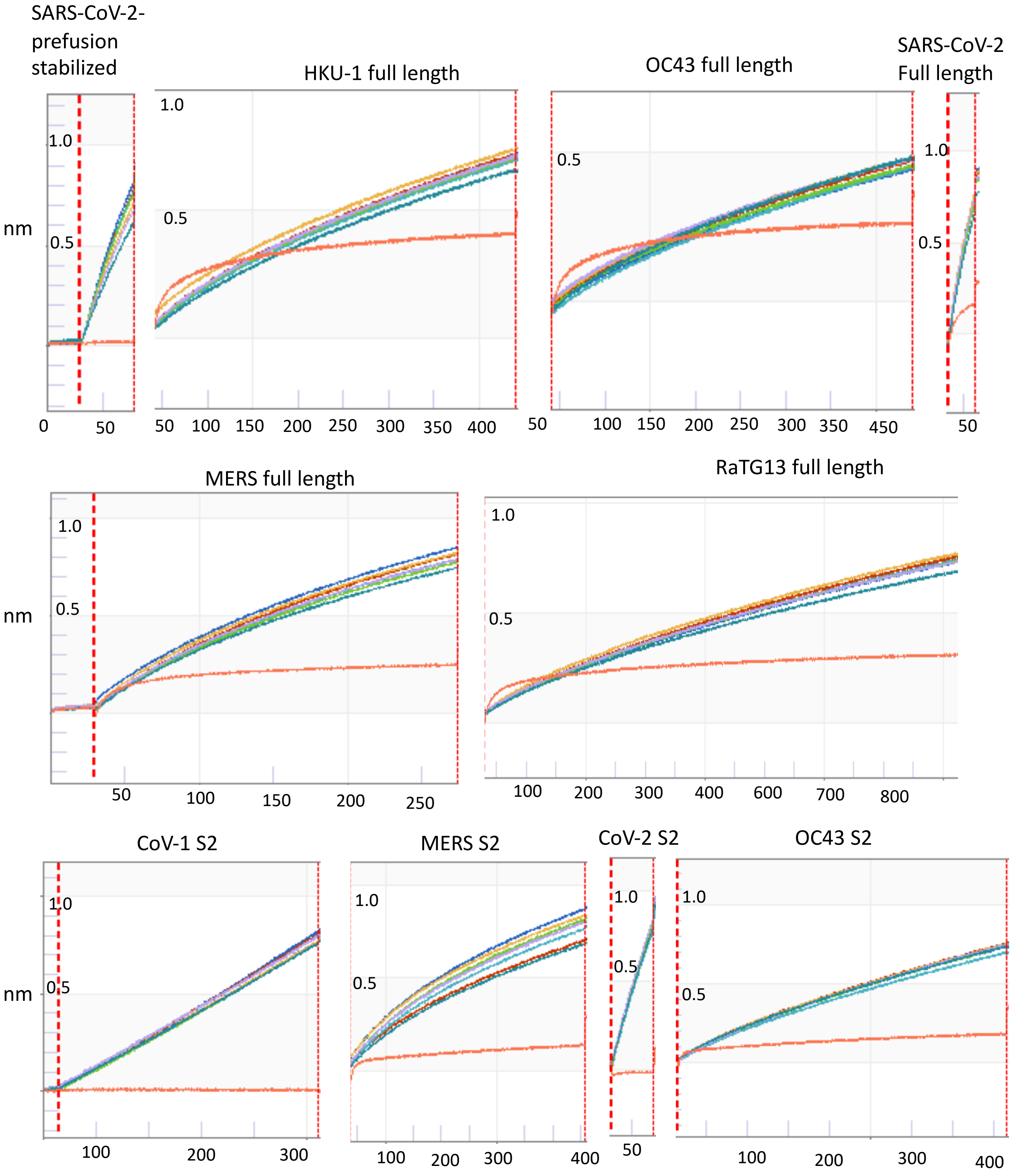
SARS-CoV-2-prefusion stabilized
HKU-1 full length
OC43 full length
SARS-CoV-2 Full length
nm
1.0
0.5
0 50
50 100 150 200 250 300 350 400
50 100 150 200 250 300 350 450
50
MERS full length
RaTG13 full length
50 100 150 200 250
100 200 300 400 600 700 800
CoV-1 S2
MERS S2
CoV-2 S2
OC43 S2
100 200 300
100 200 300 400
50
100 200 300 400

Figure S10. Representative loading sensograms for each antigen used in BLI assays.
