# Supplementary material for: Human antibody targeting of coronavirus spike S2 subunit is associated with protection mediated by Fc effector functions
Source: J Virol. 2025 Nov 12;99(12):e01523-25. doi: 10.1128/jvi.01523-25 (PMC12724371; doi:10.1128/jvi.01523-25)
Supplement: Supplemental tables — Tables S1 to S3. [file jvi.01523-25-s0002.docx]

**Supplementary Table S1. CryoEM data collection statistics.**

1871 Fab-OC43 S2 complex

| PDB ID | 9NQZ |
| --- | --- |
| **Data collection and processing** |  |
| Magnification | 92,000 |
| Voltage (kV) | 300 |
| Electron exposure (e-/Å^2^) | 54 |
| Pixel size (Å) | 1.03 |
| Symmetry imposed | C3 |
| Initial particle images (no.) | 444,705 |
| Final particle images (no.) | 109,156 |
| Map resolution (Å) | 2.60 |
| FSC threshold | 0.143 |
| Map resolution range (Å) | 2.27-5.71 |

| **Refinement** | | |
| --- | --- | --- |
| PDB ID |  |  |
| Model composition |  |  |
| Non-hydrogen atoms | 11,985 |  |
| Macromolecules | 1554 |  |
| Glycan | 0 |  |
| Rms deviations |  |  |
| Bond lengths (Å) | 0.002 |  |
| Bond angles (^o^) | 0.46 |  |
| Ramachandran plot |  |  |
| Favored (%) | 98.2 |  |
| Allowed (%) | 1.8 |  |
| Outliers (%) | 0 |  |
| Rotamer outliers (%) | 4.5 |  |
|  |  |  |
|  |  |  |
|  |  |  |
|  |  |  |
|  |  |  |
|  |  |  |
|  |  |  |
|  |  |  |
|  |  |  |
|  |  |  |
|  |  |  |

**Supplementary Table S2. X-ray crystallography data collection and refinement statistics.**

| 1871 Fab | | |
| --- | --- | --- |
| PDB ID | 9NQ3 |  |
| Data Collection |  |  |
| Space group | P 2_1_ 2_1_ 2 |  |
| Cell dimensions |  |  |
| a,b,c (Å) | 82.192, 101.333, 134.982 |  |
| α, β, γ (^o^) | 90, 90, 90 |  |
| Resolution (Å) | 70.2-2.51 (2.56-2.51) |  |
| No. molecules in ASU | 2 |  |
| No. total observations | 334,136 (25,530) |  |
| No. unique observations | 47,883 (3377) |  |
| Multiplicity | 7.0 (7.0) |  |
| R_merge_ | 0.314 (1.06) |  |
| R_pim_ | 0.129 (0.433) |  |
| Completeness (%) | 99.9 (99.6) |  |
| <I/𝛔I> | 6.1 (2.1) |  |
| CC_1/2_ | 0.984 (0.615) |  |
| Wilson B-factor | 35.8 |  |
| Refinement |  |  |
| Non-hydrogen atoms | 6644 |  |
| Macromolecules | 6622 |  |
| R_work_ | 0.215 (0.289) |  |
| R_free_ | 0.251 (0.328) |  |
| Rms deviations |  |  |
| Bond lengths (Å) | 0.004 |  |
| Bond angles (^o^) | 0.86 |  |
| Ramachandran plot |  |  |
| Favored (%) | 96.6 |  |
| Allowed (%) | 3.4 |  |
| Outliers (%) | 0 |  |
| Rotamer outliers (%) | 1.8 |  |
| B-factors (Å^2^) |  |  |
| Wilson B-factor | 35.8 |  |
| Average B-factor | 40.7 |  |
| Average macromolecules | 40.8 |  |

Statistics for the highest-resolution shell are shown in parentheses.

**Supplementary Table S3. OC43 S2 – 1871 Fab hydrogen bonding residues identified by PISA. Note: (HB) Hydrogen bond (3.7 Å cut-off)**

| Spike residue number | Residue | Interaction | BSA (Å^2^) | Fab 1871 (H-HC, L-KC) |
| --- | --- | --- | --- | --- |
| 52 | Ser^O^ | HB | 21.8 | L-Arg32^NH2^ |
| 54 | Lys^O^ | HB | 55.3 | H-Tyr109^N^ |
|  | Lys^NZ^ | HB |  | L-Asn93^O^ |
|  |  | HB |  | L-Tyr91^O^ |
| 56 | Thr^O^ | HB | 70.2 | H-Cys107^N^ |
|  |  | HB |  | H-Cys107^O^ |
|  | Thr^OG1^ | HB |  | H-Tyr109^OH^ |
| 58 | Asp^OD2^ | HB | 96.0 | H-Ser53^N^ |
|  |  | HB |  | H-Ser53^OG^ |
|  |  | HB |  | H-Ser55^N^ |
|  |  | HB |  | H-Ser54^OG^ |
|  | Asp^N^ | HB |  | H-Gly105^O^ |
| 60 | Ala^N^ | HB | 32.8 | H-Tyr57^OH^ |
| 82 | Asp^OD1^ | HB | 37.9 | H-Tyr59^OH^ |
|  |  | HB |  | L-Trp94^NE1^ |
| 89 | ThrA^O^ | HB | 60.0 | L-Arg30^NH1^ |
| 89 | ThrA^OG1^ | HB |  | L-Trp94^N^ |
| 90 | Glu^O^ | HB | 22.5 | L-Trp94^N^ |
